# AR-induced long non-coding RNA LINC01503 facilitates proliferation and metastasis via the SFPQ-FOSL1 axis in nasopharyngeal carcinoma

**DOI:** 10.1038/s41388-020-01388-8

**Published:** 2020-07-13

**Authors:** Shi-Wei He, Cheng Xu, Ying-Qing Li, Ying-Qin Li, Yin Zhao, Pan-Pan Zhang, Yuan Lei, Ye-Lin Liang, Jun-Yan Li, Qian Li, Yang Chen, Sheng-Yan Huang, Jun Ma, Na Liu

**Affiliations:** Sun Yat-sen University Cancer Center, State Key Laboratory of Oncology in South China, Collaborative Innovation Center of Cancer Medicine, Guangdong Key Laboratory of Nasopharyngeal Carcinoma Diagnosis and Therapy, No. 651 Dongfeng Road East, Guangzhou, 510060 Guangdong China

**Keywords:** Head and neck cancer, Cell migration, Prognostic markers

## Abstract

Increasing evidence indicates that long non-coding RNAs (lncRNAs) play vital roles in the tumorigenesis and progression of cancers. However, the functions and regulatory mechanisms of lncRNAs in nasopharyngeal carcinoma (NPC) are still largely unknown. Our previous lncRNA expression profiles identified that LINC01503 was overexpressed in NPC. Here, we verified that LINC01503 was highly expressed in NPC and correlated with poor prognosis. LINC01503 promoted NPC cell proliferation, migration, and invasion in vitro, and facilitated tumor growth and metastasis in vivo. Mechanistically, LINC01503 recruited splicing factor proline-and glutamine-rich (SFPQ) to activate Fos like 1 (FOSL1) transcription, and ectopic expression of FOSL1 reversed the suppressive effect of LINC01503 knockdown on NPC progression. Moreover, androgen receptor (AR)-mediated transcription activation was responsible for the overexpression of LINC01503, and AR ligand-dependent cell growth, migration, and invasion in NPC cells. Taken together, our findings reveal that AR-induced LINC01503 can promote NPC progression through the SFPQ-FOSL1 axis, which represents a novel prognostic biomarker and therapeutic target for NPC patients.

## Introduction

Nasopharyngeal carcinoma (NPC) is an epithelial malignant tumor that originates from the mucosa of the nasopharyngeal cavity [1]. NPC has a high incidence in Southeast Asia, especially in China, accounting for almost 40% of new cases worldwide [2]. Recent advances in intensity modulated radiation therapy and its combination with chemotherapy have remarkably improved the efficacy of NPC treatments, especially in the early stage [3–6]. However, more than 70% of NPC cases are diagnosed as advanced-stage disease, and local relapse and distant metastasis are two main reasons for NPC death [7–9]. Therefore, the identification of key biomarkers and characterization of its mechanisms involved in NPC recurrence and metastasis would make a significant difference on the development of individualized treatment for NPC patients.

Long non-coding RNAs (lncRNAs) are a class of RNA molecules that are longer than 200 nucleotides and have no protein-coding capacity [10]. They function as essential regulators in gene expression networks by controlling nuclear architecture, transcription, mRNA stability, translation, and posttranslational modifications [11–13]. Increasing evidence indicates that lncRNAs have many kinds of biological functions, and they play vital roles in differentiation, development, immunoregulation, and tumorigenesis [14–16]. Abnormal expression of lncRNAs has been found in multiple tumors, including NPC [17–19]. It has been reported that several dysregulated lncRNAs, such as DANCR, FAM225A, PVT1, NKILA, THOR, play critical roles in NPC development and progression [3, 20–22]. Nevertheless, only a small number of lncRNAs have been identified, and the study of the functions of lncRNAs in NPC is still in the preliminary stage. Therefore, further studies on the regulatory mechanism of lncRNAs would provide biomarkers and therapeutic targets for NPC patients.

Based on our previous lncRNA expression profile (GSE126683), we found that lncRNA LINC01503 was overexpressed in NPC tissues [3], but its biological function and regulatory mechanism involved in NPC have not been elucidated. Here, in our present study, we confirmed that LINC01503 was overexpressed in NPC and correlated with poor prognosis. Functional studies revealed that LINC01503 promoted NPC cell growth and metastasis in vitro and in vivo. Mechanistically, LINC01503 recruited splicing factor proline-and glutamine-rich (SFPQ) to activate Fos like 1 (FOSL1) transcription, which was involved in NPC progression. In addition, androgen receptor (AR)-mediated transcription activation was found to be an AR ligand-dependent response, responsible for LINC01503 overexpression in NPC. This newly identified AR-LINC01503-SFPQ-FOSL1 regulatory axis represents a novel prognostic biomarker and therapeutic target for NPC.

## Results

### LINC01503 is highly expressed in NPC and correlates with poor prognosis

**Fig. 1:**
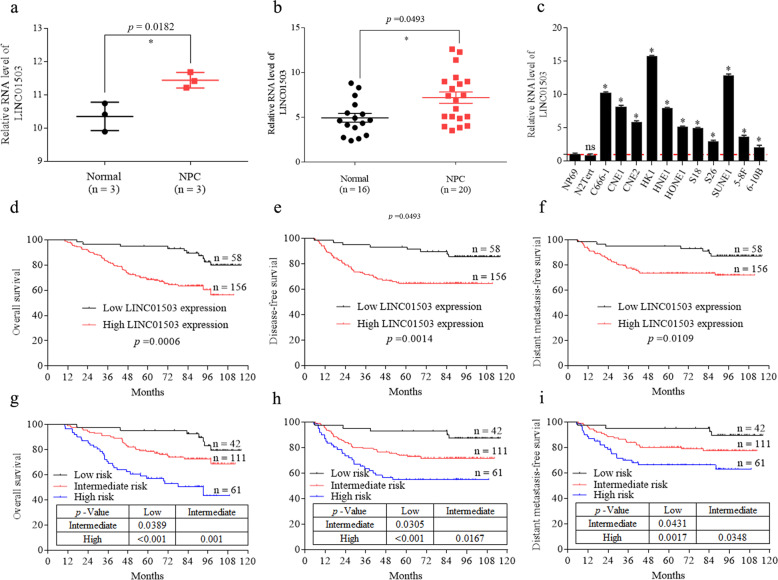
LINC01503 is highly expressed in NPC and correlates with poor prognosis. **a** LINC01503 was highly expressed in NPC tissues compared with normal nasopharynx tissues according to our previous microarray data (GSE126683; 3 paired samples). **b** Relative expression of LINC01503 in 16 normal nasopharynx and 20 NPC tissues was detected by RT-qPCR. **c** Relative expression of LINC01503 in multiple NPC and nasopharyngeal epithelial cell lines (NP69 and N2Tert) was tested by RT-qPCR. Data are presented as the mean ± SD; *p* values were calculated with Student’s *t* test; *p < 0.05; ns not significant. **d**–**f** Relative LINC01503 expression in 214 NPC tissues was tested by RT-qPCR. Kaplan–Meier analysis of overall survival (**d**), disease-free survival (**e**), and distant metastasis-free survival (**f**) in NPC patients with low LINC01503 expression (*n* = 58) and high LINC01503 expression (*n* = 156). Kaplan–Meier analysis of overall survival (**g**), disease-free survival (**h**), and distant metastasis-free survival (**i**) according to the prognostic prediction model, low risk (low LINC01503 expression and early TNM stage, *n* = 42), intermediate risk (high LINC01503 expression or advanced TNM stage, *n* = 111), and high risk (high LINC01503 expression and advanced TNM stage, *n* = 61). The *p* values were calculated with the log-rank test.

Based on our previous genome-wide lncRNA profile, LINC01503 was found to be highly expressed in NPC tissues [3] (Fig. 1a). To confirm this result, we tested the expression level of LINC01503 in 20 NPC tissues and 16 normal nasopharynx tissues by quantitative RT-PCR. The results showed that the expression of LINC01503 was obviously overexpressed in NPC tissues (Fig. 1b). In addition, LINC01503 was significantly upregulated in 11 NPC cell lines compared with the normal nasopharyngeal epithelial cells NP69 and N2Tert (Fig. 1c).

Then, we examined LINC01503 expression in a cohort of 214 paraffin-embedded tumor tissues from NPC patients to assess its clinical significance. Patients were assigned to a low (*n* = 58) or high (*n* = 156) LINC01503 expression group based on ROC analysis. Kaplan–Meier analysis showed that patients with high LINC01503 level had worse overall, disease-free, and distant metastasis-free survival than those with low LINC01503 level (Fig. 1d–f). However, there were no significant associations between LINC01503 expression and clinical characteristics (Supplementary Table 1). Multivariable Cox analysis showed that LINC01503 expression and TNM stage were independent prognostic factors (Supplementary Table 2).

We next constructed a predictive model for NPC (214 patients) prognosis by combining the LINC01503 expression and TNM stage data. The NPC patients were divided into three groups: 42 (19.62%) patients in low-risk group (low LINC01503 expression and early TNM stage), 111 (51.87%) patients in intermediate-risk group (high LINC01503 expression or advanced TNM stage), and 61 (28.50%) patients in high-risk group (high LINC01503 expression and advanced TNM stage). Kaplan–Meier curves showed that NPC patients in these three groups displayed different survival prospects (Fig. 1g-i). Summarily, these findings suggest that LINC01503 is upregulated in NPC and may serve as a biomarker for NPC prognosis.

### LINC01503 promotes NPC cell growth, migration and invasion in vitro

**Fig. 2:**
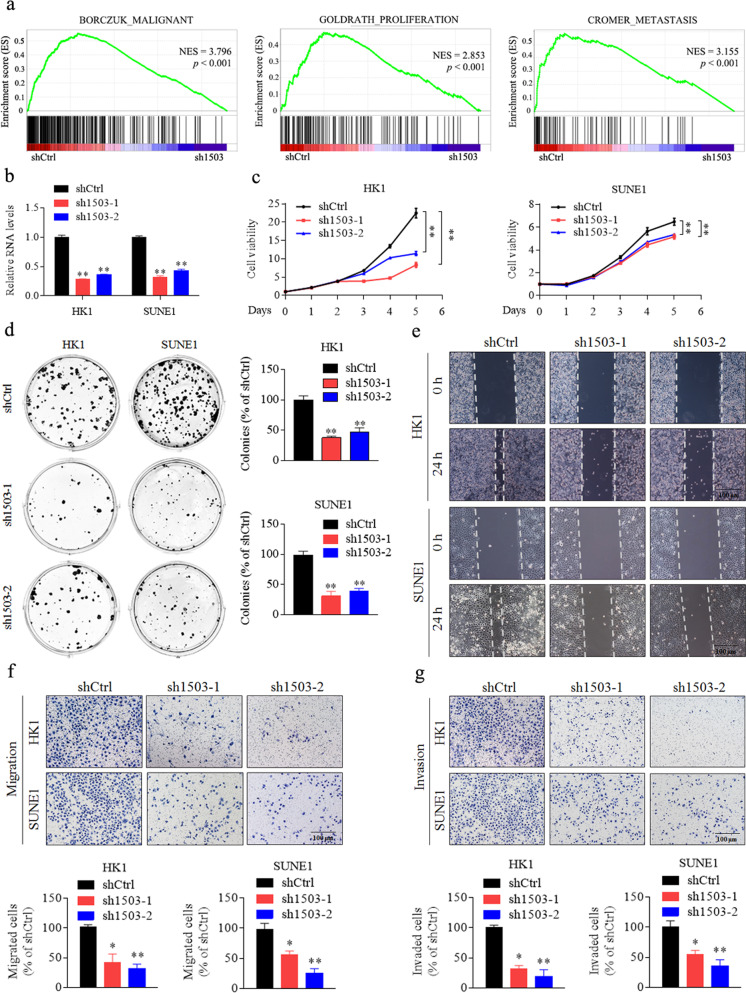
Silencing of LINC01503 inhibits NPC cell growth, migration, and invasion. **a** Malignant, proliferation-related, and metastasis-related biological functions were enriched by gene set enrichment analysis (GSEA) in HK1 cells transfected with LINC01503 shRNA (sh1503) or shCtrl. NES normalized enrichment score. FDR < 0.25, *p* < 0.001. **b** Relative expression of LINC01503 upon specific shRNA knockdown in HK1 and SUNE1 cells. **c** LINC01503 knockdown inhibited the cell growth of HK1 and SUNE1 cells as tested by CCK-8 assays. **d** LINC01503 knockdown decreased cellular survival effects as evaluated by colony formation assays. **e** LINC01503 knockdown inhibited the cellular movement ability of HK1 and SUNE1 cells as assessed by wound healing assays. Scale bar, 100 μm. **f**, **g** LINC01503 knockdown inhibited the migration and invasion ability of HK1 and SUNE1 cells as determined by Transwell assays. Scale bar, 100 μm. Data are presented as the mean ± SD; *p* values were calculated with Student’s *t* test; *p < 0.05, **p < 0.01.

To determine the potential role of LINC01503 in NPC, we specifically knock downed endogenous LINC01503 expression in HK1 cells, and then performed RNA-seq to identify the downstream genes affected by LINC01503 knockdown. The results showed that 1480 genes were decreased and 1371 genes were increased (fold change ≥1.5, *p* < 0.05). Then, GO and KEGG analyses showed that dysregulated genes were enriched in cancer-related pathways (Supplementary Fig. 1). GSEA found that the gene-sets related to malignancy, proliferation, and metastasis were negatively correlated with LINC01503 downregulation (Fig. 2a). These data confirmed that LINC01503 is extremely relevant to NPC progression.

To validate these findings, we transiently knocked down LINC01503 expression in HK1 and SUNE1 cells with two different shLINC01503 plasmids and performed in vitro functional assays (Fig. 2b). CCK-8 and colony formation assays showed that the knockdown of LINC01503 significantly suppressed NPC cell growth and proliferation (Fig. 2c, d). Wound healing and Transwell assays displayed that LINC01503 knockdown inhibited the migratory and invasive potential of NPC cells (Fig. 2e, f). In contrary, overexpression of LINC01503 promoted the malignant phenotypes of 5–8F and HONE1 cells (Supplementary Fig. 2). These results suggest that LINC01503 promotes NPC cell growth, migration, and invasion in vitro.

### LINC01503 directly interacts with SFPQ

**Fig. 3:**
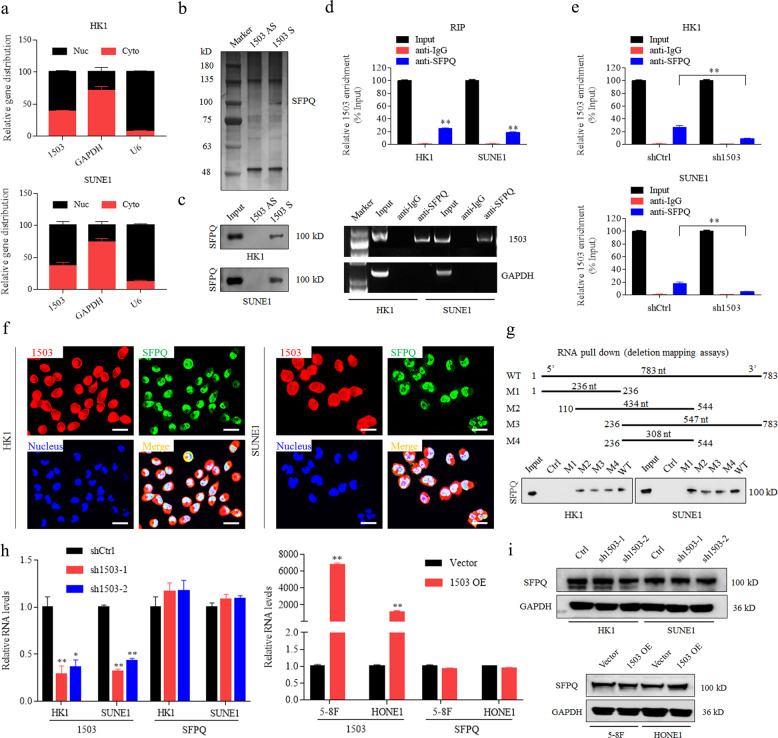
LINC01503 directly interacts with SFPQ in NPC. **a** Relative expression of LINC01503 in the cytoplasm and nucleus of HK1 and SUNE1 cells was measured by nucleocytoplasmic separation and RT-qPCR assays. **b** SFPQ was a potential interacting candidate of LINC01503. Biotin-labeled full-length LINC01503 transcript (sense) and anti-sense were incubated with HK1 cell lysates, and the enriched proteins were collected and subjected to SDS-PAGE electrophoresis and silver staining. Specific bands were excised and analyzed by mass spectrometry. **c** LINC01503 associated with SFPQ as monitored by RNA pull-down and western blot assays. **d** LINC01503 was enriched by anti-SFPQ antibody in HK1 and SUNE1 cells as performed by RNA immunoprecipitation (RIP). Then, GAPDH or enriched LINC01503 was analyzed by PCR. **e** Knockdown of LINC01503 decreased the interaction of LINC01503 with SFPQ in HK1 and SUNE1 cells as monitored by RIP assay. **f** Location of LINC01503 and SFPQ in HK1 and SUNE1 cells was visualized by RNA-FISH and immunofluorescence assays. Scale bar, 50 μm. **g** Deletion mapping assays suggested that the region 236–544 nt region in LINC01503 was required for interacting with SFPQ. **h** The RNA levels of SFPQ were not altered in either HK1 and SUNE1 cells with LINC01503 knockdown or 5–8F and HONE1 cells with LINC01503 overexpression. **i** The protein levels of SFPQ were not altered in the above (**h**) treated cells. The data are presented as the mean ± SD, and *p* values were calculated with Student’s *t* test; *p < 0.05, **p < 0.01.

To explore how LINC01503 exerts its biological function on NPC cells, we performed a nuclear-cytoplasmic RNA extraction assay and found that LINC01503 was located in both nucleus and cytoplasm of HK1 and SUNE1 cells, but especially in the nucleus (Fig. 3a). LncRNAs have been reported to exert their functions by interacting with specific proteins [23], thus, we performed an RNA pull-down assay with biotin-labeled LINC01503 sense or its antisense sequence, and then performed mass spectrometry analysis to identify LINC01503-interacting proteins (Fig. 3b). The result displayed that splicing factor SFPQ was one of the highly enriched proteins and was further verified by western blotting (Supplementary Table 3, Fig. 3c).

To further validate the physical interaction between LINC01503 and SFPQ, we performed a RIP assay with an anti-SFPQ antibody and found that LINC01503 RNA was obviously enriched (Fig. 3d). Silencing LINC01503 abolished the interaction between LINC01503 and SFPQ (Fig. 3e). Moreover, FISH combined with IF staining showed that LINC01503 was colocalized with SFPQ in the nuclei of HK1 and SUNE1 cells (Fig. 3f). Then, to determine the specific fragment of LINC01503 required for its binding with SFPQ, we constructed a series of LINC01503 truncation plasmids and conducted RNA pull-down assay. The results indicated that the 236–544 nt region of LINC01503 236–544 nt was required for its interaction with SFPQ (Fig. 3g). However, neither knockdown nor overexpression of LINC01503 affected SFPQ mRNA or protein levels (Fig. 3h, i). These results indicate that LINC01503 can directly bind to SFPQ.

### LINC01503 recruits SFPQ to activate FOSL1 transcription

**Fig. 4:**
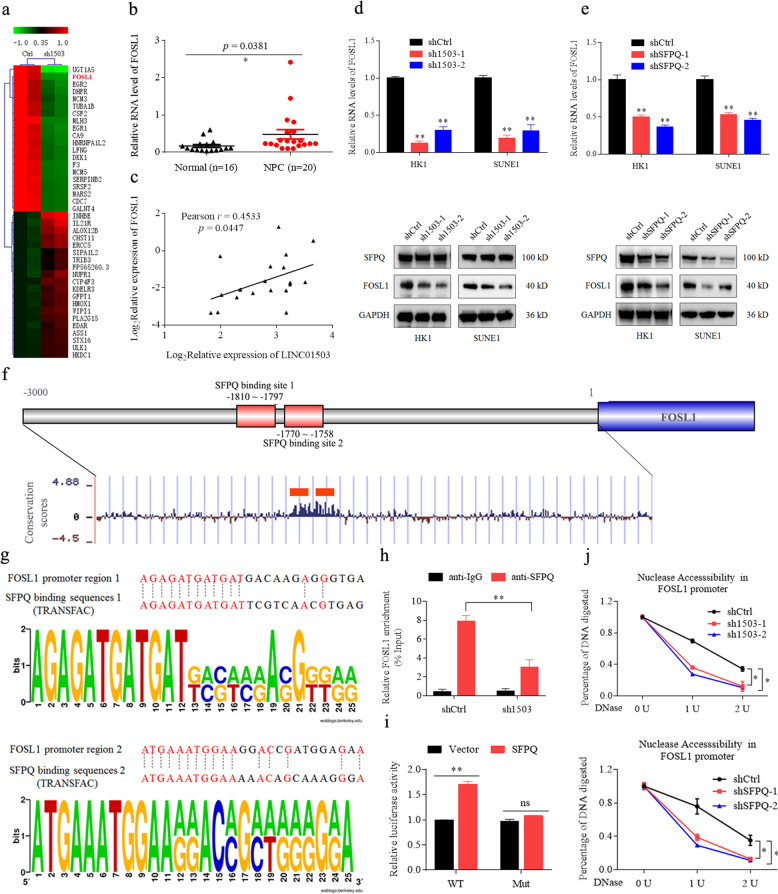
LINC01503 recruits SFPQ to activate FOSL1 transcription. **a** Heatmap of clustering of differentially expressed genes in sh1503- and shCtrl-treated HK1 cells. **b** The expression of FOSL1 was increased in 20 NPC tissues compared with 16 normal tissues as determined by RT-qPCR. **c** LINC01503 expression was positively correlated with FOSL1 mRNA expression in 20 NPC tissues. **d** The RNA and protein levels of FOSL1 were decreased in sh1503-treated HK1 and SUNE1 cells as monitored by RT-qPCR and western blot. **e** The RNA and protein levels of FOSL1 were decreased in shSFPQ-treated HK1 and SUNE1 cells as monitored by RT-qPCR and WB. **f** Schematics of the 5′ region (-3000-0 nt) of the FOSL1. There are two SFPQ-binding sites in the promoter region of FOSL1, −1810 to −1797 and −1770 to −1758 nt. **g** Comparisons of SFPQ-binding sequences and the 5′ region of the FOSL1. The SFPQ-binding sequence was predicted by TRANSFAC analysis. **h** LINC01503 knockdown inhibited the enrichment of SFPQ on the FOSL1 promoter region in HK1 cells as indicated by chromatin immunoprecipitation (ChIP). **i** SFPQ overexpression increased the luciferase activity of the FOSL1 wild-type promoter construct, but not the mutant reporter gene construct, as determined by luciferase reporter assays in HEK293T cells. **j** LINC01503 and SFPQ knockdown decreased the chromatin accessibility at the promoter of the FOSL1 gene in HK1 cells as tested by DNase I digestion assays. Data are presented as mean ± SD; *p* values were calculated with Student’s *t* test; *p < 0.05, **p < 0.01.

As a multifunctional nuclear protein, SFPQ can function as a transcriptional suppressor or activator to regulate gene expression [24]. To determine the target gene of LINC01503/SFPQ in NPC, we analyzed the RNA-seq data in HK1 cells with or without LINC01503 knockdown. We identified the top 20 upregulated and downregulated genes, of which FOSL1 attracted our attention (Fig. 4a). FOSL1 was overexpressed and positively correlated with LINC01503 expression in NPC tissues (Fig. 4b, c). Importantly, either knockdown of LINC01503 or SFPQ could decrease FOSL1 mRNA and protein levels (Fig. 4d, e).

Then, we predicted that there were two SFPQ-binding sites located in the promoter region of the FOSL1 gene using the Transfac and Weblogo programmes (Fig. 4f, g). Chip-PCR demonstrated that the enrichment of SFPQ at the promoter region of FOSL1 could be significantly abolished by LINC01503 knockdown (Fig. 4h). Furthermore, a luciferase reporter assay illustrated that overexpression of SFPQ significantly increased the luciferase activity of the FOSL1 wild-type promoter construct, but not the mutant reporter construct (Fig. 4i). Finally, DNase I digestion assay showed that LINC01503 or SFPQ knockdown significantly inhibited the chromatin accessibility in the FOSL1 promoter locus (Fig. 4j). These findings demonstrate that LINC01503 can recruit SFPQ to activate FOSL1 transcription activity and increase its expression.

### FOSL1 is responsible for LINC01503/SFPQ-mediated NPC progression

**Fig. 5:**
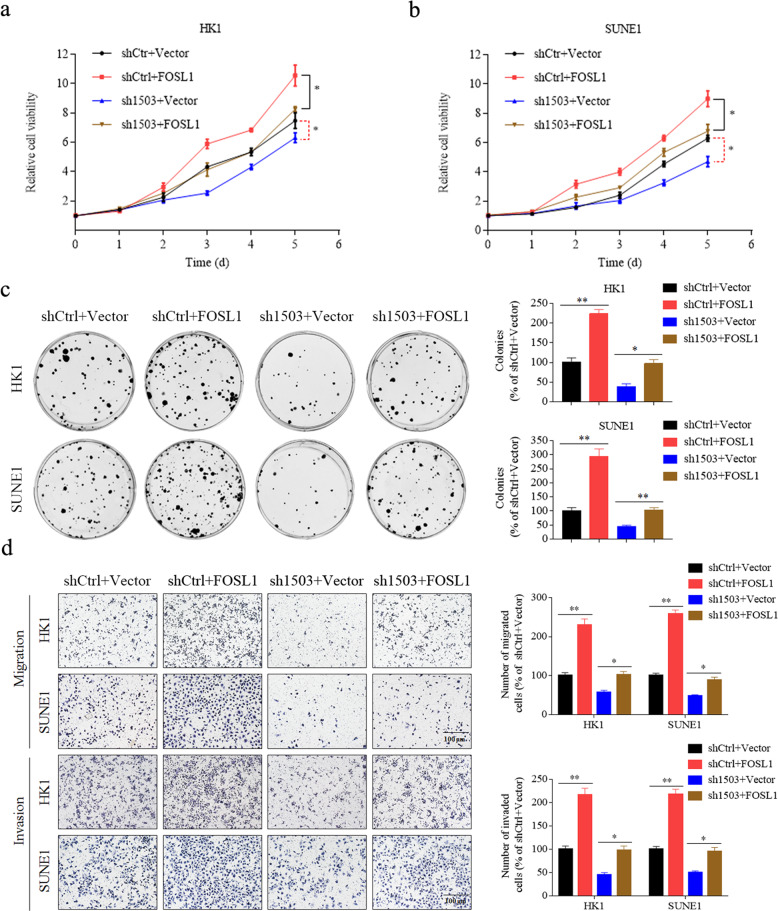
FOSL1 is responsible for LINC01503/SFPQ-mediated NPC progression. **a**, **b** Knockdown of LINC01503 restrained cell proliferation while FOSL1 overexpression rescued it in HK1 and SUNE1 cells as demonstrated by CCK-8 assays. **c** FOSL1 overexpression increased the cellular survival of LINC01503 knockdown HK1 and SUNE1 cells as evaluated by colony formation assays. **d** Knockdown of LINC01503 inhibited cell migration and invasion while FOSL1 overexpression rescued it in HK1 and SUNE1 cells as determined by Transwell assays. Data are presented as the mean ± SD; *p* values were calculated with student’s *t* test; *p < 0.05, **p < 0.01.

To determine whether LINC01503/SFPQ promotes NPC progression by activating FOSL1, we introduced a FOSL1 overexpression or empty vector in HK1 and SUNE1 cells with stable knockdown of LINC01503 (sh1503) or its control (shCtrl), as monitored by quantitative RT-PCR and western blotting (Supplementary Fig. 3). CCK-8 and colony formation assays showed that the inhibitory effect of LINC01503 knockdown on cell growth and proliferation was rescued by overexpressing FOSL1 in HK1 and SUNE1 cells (Fig. 5a–c). Moreover, transwell assays showed that overexpression of LINC01503 reversed the inhibitory effect of LINC010503 knockdown on NPC cell migratory and invasive capacities (Fig. 5d). Our results illustrate that LINC01503 promotes NPC progression by activating FOSL1.

### Knockdown of LINC01503 inhibits NPC tumor growth and metastasis in vivo

**Fig. 6:**
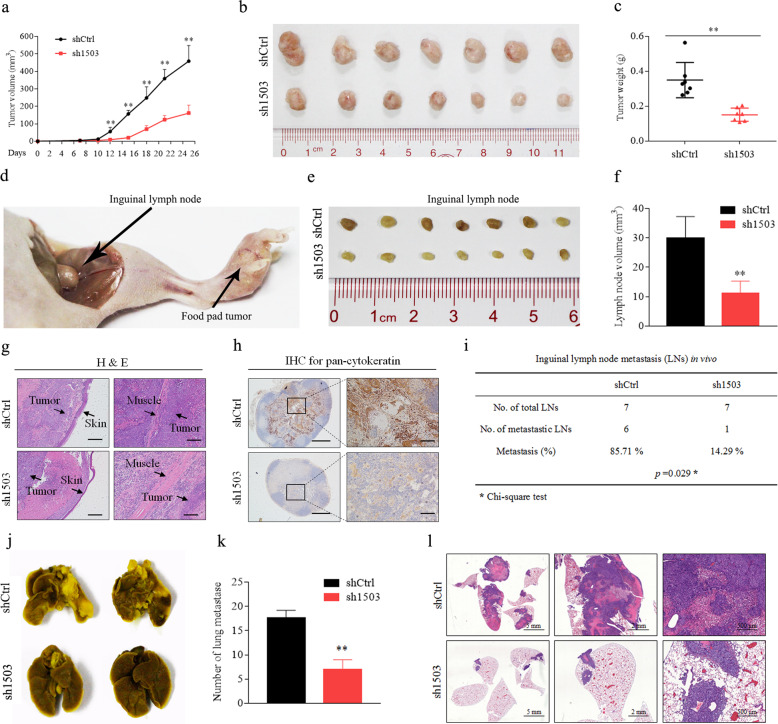
Knockdown of LINC01503 inhibits NPC tumor growth and metastasis in vivo. **a** Tumor growth curves of shCtrl and sh1503 HK1 cells in xenograft tumor growth model. **b** Representative image of xenograft tumors. **c** Tumor weight was smaller in the sh1503 group than in the shCtrl group. **d** Representative image of primary tumor in foot pads and metastatic inguinal lymph nodes in the inguinal lymph node metastasis model. **e** Representative image of the inguinal lymph node. **f** Inguinal lymph node volume was smaller in the sh1503 group. **g** H&E staining of foot pad primary tumors. **h** Pan-cytokeratin staining for inguinal lymph nodes as tested by IHC assay. **i** The metastatic ratio of inguinal lymph nodes was smaller in the sh1503 group. **j** Representative images of metastatic nodules on the lungs in the lung metastatic colonization model. **k** The number of metastatic nodule in lungs was lower in the sh1503 group. **l** H&E staining showed that the metastatic nodules were fewer and smaller in the sh1503 group. Data are presented as the mean ± SD; *p* values were calculated with Student’s *t* test; *p < 0.05, **p < 0.01.

To determine the role of LINC01503 in NPC growth and metastasis in vivo, we firstly constructed a tumor growth model by injecting HK1 cells stably expressing shLINC01503 or its scramble control into the dorsal flank of nude mice. As shown in Fig. 6a, knockdown of LINC01503 significantly delayed the tumor growth. The formed tumors in the shLINC01503 group were smaller than those in the control group (Fig. 6b), which was confirmed by the tumor weight data (Fig. 6c). FISH combined IHC assays validated that knockdown of LINC01503 resulted in a decrease in FOSL1 and Ki67 expression, while did not affect the expression of SFPQ in the formed tumors (Supplementary Fig. 4).

Then, we established an inguinal lymph node metastasis model by injecting HK1 cells stably expressing shLINC01503 or its scramble control into the foot pads of nude mice (Fig. 6d). The inguinal lymph nodes were smaller and their weight was lower in the shLINC01503 group (Fig. 6e, f). H&E staining showed that tumors in the LINC01503 knockdown group were less aggressive, with an impaired invasion into the skin and muscle (Fig. 6g). Furthermore, the inguinal lymph node metastatic ratio was remarkably lower in the shLINC01503 group than that in the shCtrl group, as determined by the pan-cytokeratin-positive tumor cells (Fig. 6h, i).

Finally, we constructed a lung metastatic colonization model by injecting HK1 cells stably expressing shLINC01503 or its scramble control into the tail veins of nude mice. We found that there were fewer metastatic nodules on the lung surfaces were fewer in the shLINC01503 group than in the control group (Fig. 6j). The statistical analysis of lung metastatic nodules amount also verified the previous results (Fig. 6k). Moreover, H&E staining showed that the metastatic nodules were fewer and smaller in the shLINC01503 group than that in shCtrl group (Fig. 6l). Collectively, these data suggest that LINC01503 promotes NPC tumor growth and lung metastasis in vivo.

### AR activates LINC01503 transcription to increase its expression in NPC

**Fig. 7:**
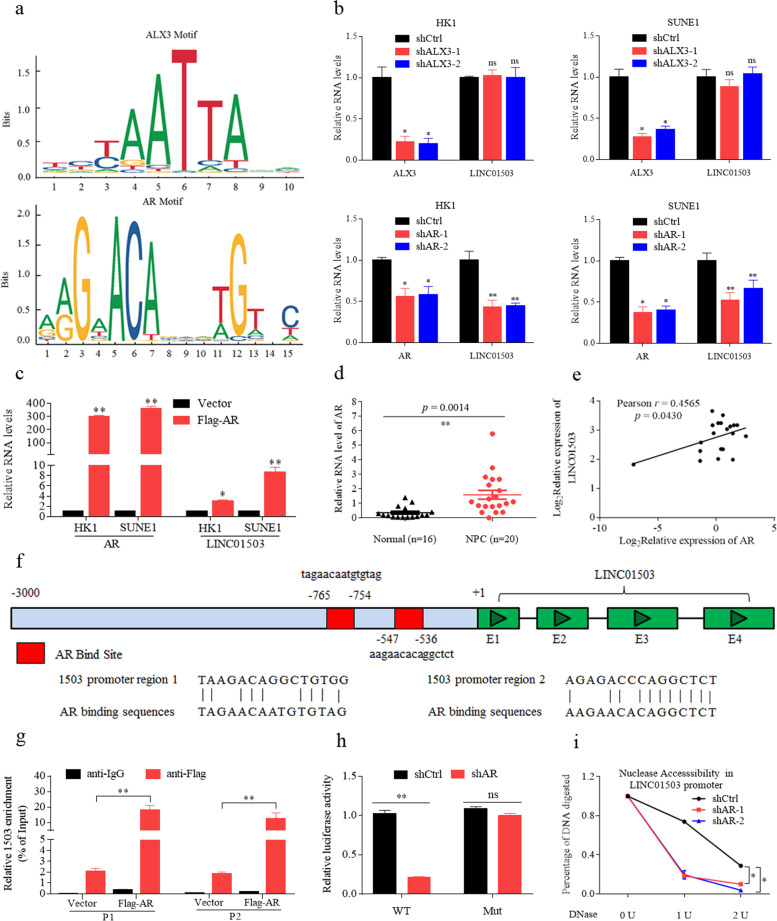
AR activates LINC01503 transcription to increase its expression in NPC. **a** The DNA-binding motif of ALX homeobox 3 (ALX3) and androgen receptor (AR) on the LINC01503 promoter was analyzed by JASPAR software. **b** Knockdown of ALX3 did not influence the expression of LINC01503, while knockdown of AR inhibited the expression of LINC01503 in HK1 and SUNE1 cells as detected by RT-qPCR. **c** Overexpression of AR increased the expression of LINC01503 in HK1 and SUNE1 cells. **d** The expression of AR was higher in 20 NPC tissues than in 16 normal nasopharynx tissues as determined by RT-qPCR. **e** AR mRNA expression was positively correlated with LINC01503 expression in 20 NPC tissues. **f** The potential binding sites of AR at the promoter region of LINC01503. **g** Overexpression of AR enhanced its enrichment at the LINC01503 promoter region in HK1 cells as monitored by ChIP-qPCR assay. **h** Knockdown of AR increased the luciferase activity of the LINC01503 wild-type promoter construct, but not the mutant reporter gene construct, as determined by luciferase reporter assays in HEK293T cells. **i** AR knockdown decreased the chromatin accessibility at the LINC01503 promoter in HK1 cells as tested by DNase I digestion assays. Data are presented as the mean ± SD; *p* values were calculated with Student’s *t* test; *p < 0.05, **p < 0.01.

To explore the potential mechanism of high level of LINC01503 in NPC, we analyzed the promoter of LINC01503 by JASPAR software and predicted two transcription factors, ALX homeobox 3 (ALX3) and AR (Fig. 7a; Supplementary Table 4). Next, we found that knockdown of AR obviously decreased LINC01503 expression in HK1 and SUNE1 cells, while knockdown of ALX3 did not (Supplementary Fig. 5a; Fig. 7b). Moreover, overexpression of AR increased the RNA expression of LINC01503 (Supplementary Fig. 5b; Fig. 7c). Importantly, AR was upregulated in NPC and it was positively correlated with the expression of LINC01503 (Fig. 7d, e).

Then, we predicted two high affinity binding sites in the promoter region of LINC01503 using JASPAR software (Fig. 7f). Interestingly, ChIP-PCR showed that ectopic expression of AR significantly enhanced its enrichment at the promoter region of LINC01503 (Fig. 7g). Moreover, we found that knockdown of AR significantly reduced the luciferase activity of the wild-type LINC01503 promoter construct, but not the mutant reporter construct, as demonstrated by the luciferase reporter assay (Fig. 7h). Finally, we observed that AR knockdown significantly impaired the chromatin accessibility in the LINC01503 promoter locus via a DNase I digestion assay (Fig. 7i). These results suggest that AR activates LINC01503 transcription in NPC.

**Fig. 8:**
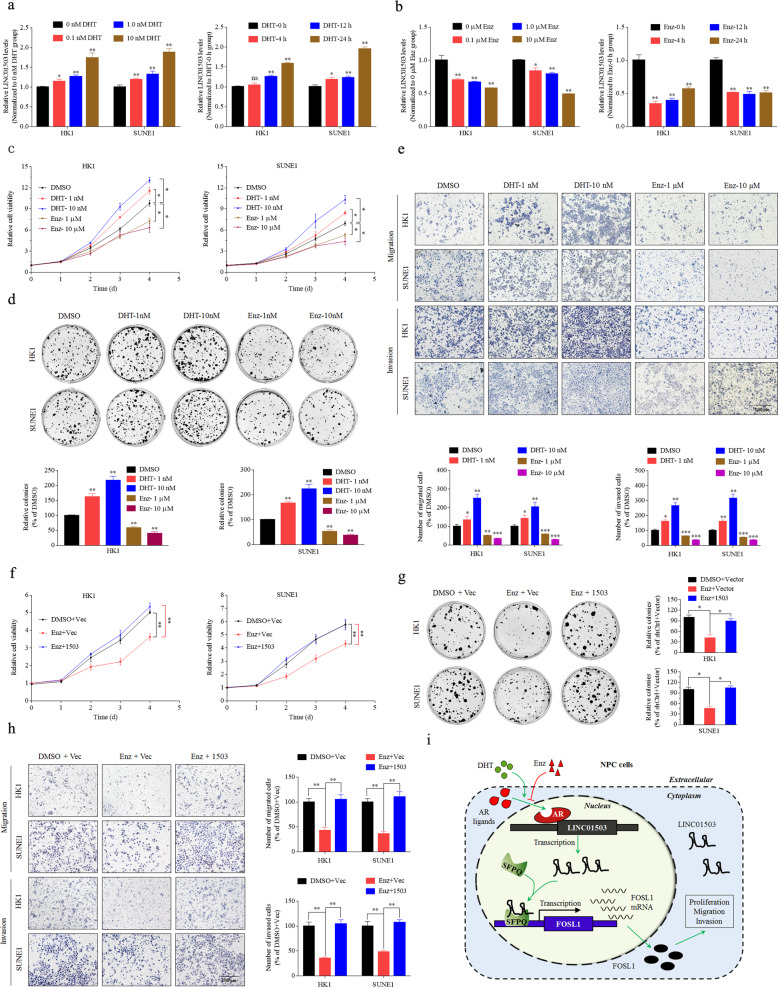
AR-mediated LINC01503 promotes the NPC malignant phenotype in an AR ligand-dependent manner. **a** The expression of LINC01503 showed dose-dependent and time-dependent activation after exposure to the AR agonist dihydrotestosterone (DHT) in HK1 and SUNE1 cells. **b** The expression of LINC01503 showed a dose-dependent and time-dependent inhibition at the presence of the AR antagonist enzalutamide (Enz) in HK1 and SUNE1 cells. **c**–**e** HK1 and SUNE1 cells displayed a dose-dependent response of cell proliferation, migration and invasion after exposure to DHT or Enz. **f**–**h** Enz-inhibited cell growth, migration and invasion could be restored by LINC01503 overexpression. **i** A graphical abstract of the LINC01503 function and mechanism in NPC. In this model, high enrichment of AR increases its occupation at the promoter region of LINC01503, which could be inhibited by Enz and be activated by DHT; and thus stimulating LINC01503 upregulation in NPC. Then, LINC01503 recruits SFPQ enriching its presence at the promoter region of FOSL1 and activating its transcription, finally promoting cell proliferation, migration, and invasion.

To further clarify the AR ligand-dependent activation of LINC01503, we performed AR response and inhibition assays. The results suggested a time-dependent and dose-dependent activation of LINC01503 during AR agonist dihydrotestosterone (DHT) treatment and a time-dependent and dose-dependent inhibition of LINC01503 at the presence of antagonist enzalutamide (Enz) (Fig. 8a, b). Interestingly, DHT treatment resulted in the activation of cell proliferation, migration, and invasion abilities, while AR treatment caused an inhibition of cell proliferation, migration, and invasion abilities of HK1 and SUNE1 cells (Fig. 8c–e), simultaneously accompanied by activation or inhibition of the expression of LINC01503 and its target FOSL1 expression (Supplementary Fig. 5c, d). Next, we examined the impact of LINC01503 expression impacts on the Enz-inhibited NPC cell malignant phenotype, and found that Enz could suppress the expression of LINC01503 and its target FOSL1 (Supplementary Fig. 5e). Importantly, we found that Enz-inhibited cell growth, migration, and invasion could be reversed by increasing LINC01503 expression in both HK1 and SUNE1 cells (Fig. 8f–h). These results verified that the AR antagonist Enz may inhibit NPC malignant progression through blocking upstream LINC01503 transcription.

## Discussion

Accumulating evidence indicates that lncRNAs function as critical regulators of various disease processes, including the initiation and progression of tumors [16]. By analyzing our previous lncRNA expression profile results, we found that LINC01503 was overexpressed in NPC. However, the role and mechanism of LINC01503 in NPC are still not elucidated. In this study, we first validated that LINC01503 was highly expressed in NPC tissues and cell lines. We further detected the LINC01503 expression in a set of 214 paraffin-embedded tumors from NPC patients to assess its clinical significance. Survival analysis showed that high LINC01503 expression was correlated with poor overall, disease-free and distant metastasis-free survival. A prognostic model combining LINC01503 expression and TNM stage could predict patient prognosis, which would guide more personalized therapy for NPC patients.

LINC01503 is a long intergenic non-protein-coding RNA, and it is located on 9q34.11. LINC01503 was first reported as a squamous cell carcinoma (SCC)-specific lncRNA of the esophageal and head and neck SCCs in 2018, and it was found to promote cell proliferation, migration, and tumor growth [25]. Several studies subsequently reported that LINC01503 was overexpressed and could promote tumorigenesis and progression in colorectal cancer [26], glioma [27], and cholangiocarcinoma [28]. Here, we performed RNA-seq followed by GSEA to explore the potential role of LINC01503 in NPC. The results showed that the gene-sets related to proliferation and metastasis were negatively correlated with LINC01503 knockdown. Then, in vitro and in vivo functional studies confirmed that LINC01503 promoted NPC proliferation and metastasis, which improving our understanding of LINC01503 as an oncogenic lncRNA in NPC.

It is known that lncRNAs can regulate gene expression by interacting with chromatin [11]. For instance, lncRNA FEZF1-AS1 suppresses p21 expression to promote gastric cancer proliferation via recruiting LSD1 to the p21 promoter and inducing its H3K4me2 demethylation [29]. LncRNA TCF7 induces TCF7 transcription through recruiting the SWI/SNF complex to its promoter, ultimately activating WNT signaling to inhibit liver cancer stem cell self-renewal [30]. LncRNA DLEU1 recruits SMARCA1 to the KPNA3 promoter and activates its transcription, thereby promoting the proliferation and migration of colorectal cancer [31]. Correspondingly, we found that LINC01503 promoted FOSL1 transcription via recruiting SFPQ at the promoter of FOSL1, thereby promoting NPC proliferation and metastasis. SFPQ, a multifunctional nuclear protein, is reported to be involved in various nuclear processes, including splicing, DNA repair, RNA transport, and transcriptional regulation [32]. SFPQ functions as a repressor or activator during the transcriptional regulation [24, 33, 34]. Here, SFPQ levels were enriched at the FOSL1 promoter and induced its transcriptional activity in NPC. FOSL1 belongs to the AP-1 transcription factor family and can stimulate tumor cell proliferation and metastasis [35, 36]. In this study, FOSL1 promoted NPC cell proliferation, migration and invasion, and ectopic expression of FOSL1 reversed the inhibitory effect of LINC01503 knockdown in NPC.

Most recently, abnormal expression of lncRNAs have been demonstrated to be regulated by transcription factors [37, 38]. It has been reported that AR, a transcription factor acting as a steroid hormone, can regulate the expression of multiple lncRNAs [39]. In prostate cancer, AR activated the expression of several lncRNAs, such as ARLNC1, PCGEM1, and SOCS2-AS1, via binding to the androgen response element (ARE) sites in the lncRNA locus and promoting tumor progression [39–41]. In embryonic neurogenesis, AR binds at ARE sites in lncRNA Sox2OT, and induces RNA polymerase II-dependent Sox2OT expression [42]. An AR mediated lncRNA ZEB1-AS1 can promote cell stemness, proliferation, and invasion of cholangiocarcinoma [43], while AR inhibited lncRNA TMPO-AS1 can facilitate cell proliferation and migration, and correlate with poor prognosis in prostate cancer [44]. In this study, AR facilitated lncRNA LINC01503 transcriptional activity via enriching at its promoter region in NPC. As an AR inhibitor, Enz can reduce the proliferation and death of prostate cancer cells [45, 46], and it also can block the malignant progression caused by AR-mediated LINC01503 in NPC, which may provide a potential drug target for individualized treatment of NPC patients.

In conclusion, our study demonstrated that LINC01503 was overexpressed in NPC, and correlated with poor prognosis. AR-induced LINC01503 functioned as an oncogenic lncRNA during NPC tumorigenesis and progression through recruiting SFPQ to the FOSL1 promoter and activating its transcription; interestingly, the entire pathway could be blocked by the AR ligand antagonist Enz or activated by the AR ligand agonist DHT (Fig. 8i). Therefore, the inhibition of LINC01503 expression and the development of AR ligand antagonists might potentially be a targeted therapeutic strategy for NPC patients.

## Materials and methods

### Clinical samples

Twenty freshly-frozen NPC and 16 normal nasopharynx tissues were obtained from Sun Yat-sen University Cancer Center (Guangzhou, China). In addition, 214 formalin-fixed paraffin-embedded NPC specimens were also obtained from Sun Yat-sen University Cancer Center from January 2006 to December 2009. All methods and strategies involving human samples were conducted according to the guidelines of the Institutional Ethical Review Boards of the Sun Yat-sen University Cancer Center (GZR2017-059) and written informed consent was obtained from all patients. The clinicopathological characteristics were collected and the median follow-up time of all patients was 83.8 months (range 10.2–115.9 months).

### Cell culture

Human normal nasopharyngeal epithelial cell lines, NP69 and N2-Tert, were maintained in keratinocyte/serum-free medium (Gibco, Grand Island, NY, USA). Human NPC cell lines, C666-1, CNE1, CNE2, HK1, HNE1, HONE1, SUNE1, 5–8F and 6–10B, were cultured in RPMI 1640 medium (Gibco) containing 10% fetal bovine serum (FBS; Gibco); S18, S26 and HEK293T cells were cultured in DMEM medium (Gibco) supplemented with 10% FBS. Cells were incubated at 37 °C in humidified incubator with 5% CO_2_ atmosphere.

### RNA extraction, reverse transcription and quantitative PCR (RT-qPCR)

Total RNA from cells was isolated using TRIzol Reagent (Life Technologies, Carlsbad, CA, USA), and from paraffin-embedded tissues was using QIAGEN FFPE RNeasy kit (Qiagen GmbH, Hilden, Germany). Nuclear and cytoplasmic RNA was purified using the NE-PER Nuclear and Cytoplasmic Extraction Reagents (Invitrogen, Grand Island, NY, USA). The cDNAs were synthesized using reverse transcriptase (Promega, Madison, WI, USA). Then, quantitative PCR reactions were performed by SYBR^®^ Select Master Mix (Invitrogen). All primers are listed in Supplementary Table 5.

### Plasmid construction, transfection and stable cell line generation

Specific shRNAs against LINC01503, SFPQ, FOSL1, ALX3, and AR were designed by the BLOCK-iT^™^ RNAi Designer (http://rnaidesigner.thermofisher.com) and then synthesized according to sequences shown in Supplementary Table 5. All shRNA sequences were cloned into the pLKO.1 vector (Addgene, Cambridge, MA, USA). The full-length sequences of LINC01503, SFPQ, FOSL1, and AR were amplified and cloned into the pHAGE-6tag-puro vector (Addgene). The wild type and mutant LINC01503 and FOSL1 promoters were cloned into the pGL3 vector (Addgene). Specific primers were listed in Supplementary Table 5.

All of the plasmids were confirmed by DNA sequencing, transiently transfected into NPC cells with Lipofectamine 2000/3000 reagents (Invitrogen), and the transfected cells were then harvested for assays 48 h later. The shLINC01503 (sh1503) or its scramble control (shCtrl) plasmids were co-transfected into HEK293T cells with the lentivirus packaging plasmids pMD2G and psPAX2 (Addgene). After 48 h incubated, the lentivirus supernatant was collected and used to infect HK1 and SUNE1 cells, and stably transfected cells were selected and maintained using puromycin.

### RNA sequencing and bioinformatics analysis

Total RNA was isolated from HK1 cells transfected with sh1503 or shCtrl, and used for sequencing library construction. Then, the purified libraries were loaded on cBot (Illumina, San Diego, CA, USA) to generate a cluster and sequenced on the NovaSeq 6000 (Illumina) platform at the Sinotech Genomics Co., Ltd (Shanghai, China). Differentially expressed genes (fold change ≥1.5, *p* < 0.05) were identified, and subjected to Gene Oncology (GO) and Kyoto Encyclopedia of Genes and Genomes (KEGG) pathway analyses using the DAVID software (https://david.ncifcrf.gov/). Furthermore, gene set enrichment analysis (GSEA) was performed to identify functional gene-sets associated with LINC01503 knockdown using GSEA software (version 3.0, www.broadinstitute.org/gsea/).

### CCK-8 and colony formation

For the CCK-8 assay, 1 × 103 cells were incubated in 96-well plate (NEST Biotechnology, Wuxi, China). After cells attachment, 10 μl CCK-8 was added to each well and incubated for 2 h, and then the absorbance value at 450 nm was measured using a spectrophotometric plate reader (BioTek ELX800, USA) at given gradient time (1, 2, 3, 4 and 5 day). For the colony formation assay, 600 cells were incubated in 6-well plate for ~7–14 days. Cell colonies were washed, fixed, stained, and counted.

### Wound healing and transwell assays

For the wound healing assay, cells were incubated to near confluence in 6-well plate, and starved in serum-free medium for 24 h. Artificial wounds were scratched on the monolayers and then images were captured at 0 and 24 h. For transwell migration and invasion assays, 5 × 10^4^ or 1 × 10^5^ cells were resuspended in serum-free medium and seeded into the upper Transwell Chamber (Costar, Cambridge, MA, USA), which was covered with or without Matrigel (BD Biosciences, San Diego, CA, USA). Meanwhile, medium containing 15% FBS was added to the lower chamber, and incubated for 6 h or 24 h. The migrated and invaded cells were fixed, stained, and counted under an inverted microscope.

### RNA pull-down, mass spectrometry, and RNA immunoprecipitation (RIP)

The full-length sense and anti-sense RNA of LINC01503 and its fragments were transcribed in vitro using the MEGAscript^™^ T7 Transcription Kit (Thermo Fisher Scientific, Waltham, MA, USA), and biotin-labeled with the Pierce^™^ RNA 3′ End Desthiobiotinylation Kit (Thermo Fisher Scientific). Specific primers are listed in Supplementary Table 5. The pull-down assays were performed by incubating biotin-labeled RNA with cell lysates using the Magnetic RNA-Protein Pull-Down Kit (Thermo Fisher Scientific). The bound proteins were subjected for Mass spectrometry (FitGene Biotechnology, Guangzhou, China), and western blotting.

For RNA immunoprecipitation (RIP), cell lysates were incubated with Protein A/G Plus Agarose (Santa Cruz Biotechnology, Santa Cruz, CA, USA) at 4 °C for 2 h, and then with 2 μg antibody of anti-SFPQ (Proteintech, Wuhan, China) or rabbit anti-IgG (Sigma-Aldrich, Saint Louis, MO, USA) at 4 °C for 2 h. The immunoprecipitated RNAs were purified for RT-qPCR and the primers are listed in Supplementary Table 5.

### Immunofluorescence and in situ hybridization

The Cy3-tagged LINC01503 fluorescence in situ hybridization probe was purchased from Ribo Bio (Guangzhou, China). After fixation and permeabilization, cells were incubated with the LINC01503 probe at 42 °C for 12–16 h, and then with anti-SFPQ antibody (Proteintech) at 4 °C overnight. Then, the cells were stained with Alexa Fluor^®^ 488 donkey anti-rabbit IgG (H + L) antibody (Life Technologies), and the nucleus was visualized using Hoechst 33342 (Invitrogen). The digoxin-labeled LINC01503 probe (Supplementary Table 5) was purchased from the Boster Biological Technology (Wuhan, China), and the in situ hybridization for tissues was performed according to the manufacturers’ instructions. The images were captured using NIKON ECLIPSE confocal microscope (Niko, Tokyo, Japan).

### Western blotting analysis

Total protein was extracted using RIPA lysis buffer, subjected to SDS-PAGE gel and then transferred to PVDF membrane (Merck Millipore, Billerica, MA, USA). The membrane was incubated with primary antibody: anti-SFPQ (1:1000, Proteintech), anti-FOSL1 (1:1000, Proteintech), anti-AR (1:1000, ABclonal, Wuhan, China), anti-Flag (1:2000; Abcam, Cambridge, MA, USA), anti-GAPDH (1:5000; Cell Signaling Technology, Danvers, MA, USA) or anti-Tubulin (1:5000; Woburn, MA, USA). Then were incubated with secondary antibodies, and detected using Chemiluminescence instrument (Bio-Rad, Hercules, CA, USA). Full unedited western blot gels are displayed in Supplementary Fig. 6.

### Chromatin immunoprecipitation (ChIP) assay

The ChIP assay was performed using the Pierce^™^ Magnetic ChIP Kit (Thermo Fisher Scientific) according to the manufacturers’ instructions as previously described [47]. The purified short clip DNA was detected using specific FOSL1 or LINC01503 promoter region primers as monitored by qPCR. The primers are listed in Supplementary Table 5.

### Luciferase activity assay

The SFPQ plasmid together with FOSL1 promoter region wild type or mutant construct or the AR plasmid together with LINC01503 promoter region wild type or mutant constructs were co-transfected into HEK293T cells. Each transfection group was co-transfected with Renilla plasmid. Luciferase activity was monitored by the Dual-Luciferase Reporter Assay Kit (Promega) according to the manufacturers’ guidelines, and relative firefly luciferase activity was normalized to the Renilla luciferase activity.

### DNase accessibility assay

Total DNA was extracted using the TIANamp Genomic DNA Kit (Tiangen Biotech, Beijing, China). Then, 5 μg of DNA was digested using RNase-free DNase I (Promega) at 37 °C for 5 min, and the reaction was stopped with DNase stop buffer. DNA was extracted and measured by qPCR using the same primers for ChIP-PCR as previously described [30].

### AR inhibition and response assays

For the dose-dependent assay, HK1 and SUNE1 cells were incubated with 10% FBS 1640 medium by adding various doses of dihydrotestosterone (DHT, Meilun Biotechnology Co., Ltd, Dalin, China), enzalutamide (Enz, Selleckchem, Houston, TX, USA) or DMSO (control) for 24 h and then total RNA was extracted for RT-qPCR. For the time-dependent assay, cells were treated with an appropriate concentration of DHT, Enz, or DMSO (control) for 0, 4, 12, and 24 h respectively, and then total RNA were extracted for RT-qPCR.

HK1 and SUNE1 cells were treated with Enz or DHT and DMSO (control) for 12 h, and then the cells were digested for using in CCK-8, colony formation, and transwell assays. For AR inhibition reverse assays, HK1 and SUNE1 cells were treated with an appropriate dose of Enz or DMSO (control) for 12 h, and then transfected with LINC01503 overexpression or vector (control) plasmid for 24 h. Then, the cells were digested for using in CCK-8, colony formation and transwell assays. Total RNA or proteins of each treatment group were extracted and subjected to RT-qPCR or WB assays.

### In vivo tumor xenograft models

Male BALB/c nude mice (4–5 weeks old, 18–20 g) were obtained from the Charles River Laboratories (Beijing, China). All of the animal experimental protocols were approved by the Institutional Animal Care and Use Ethics Committee of Sun Yat-sen University Cancer Center (L102012018110G). For tumor growth model, 1 × 10^6^ HK1 cells stably expressing sh1503 or shCtrl were subcutaneously injected into the underarm of mice (*n* = 7 per group), and the tumor size was measured. After 30 days, the mice were sacrificed and the tumors were dissected and weighed. Tumor sections were subjected to in situ hybridization and immunohistochemistry (IHC) analysis for LINC01503 and FOSL1 expression.

For the inguinal lymph node metastasis model, 3 × 10^6^ HK1 cells stably expressing sh1503 or shCtrl were injected into the foot pad of mice (*n* = 7 per group). After 40 days, the mice were sacrificed, and the foot pad tumor and inguinal lymph node were detached for H&E and IHC staining (anti-pan-cytokeratin antibody, Thermo Fisher Scientific). For the lung metastasis model, 1 × 10^6^ SUNE1 cells stably expressing sh1503 or shCtrl were injected into the tail vein of mice (*n* = 8 per group). After 2 months, mice were sacrificed and lung tissues were detached for H&E staining.

### Statistical analyses

All experiments in this subject were performed in triplicate. All statistical analyses were performed using the SPSS 22.0 software (IBM, Chicago, IL, USA), and all of the data are presented as the mean ± SD. Student’s *t* test and chi-square test were used to compare differences between groups. Receiver operating characteristic (ROC) curve was used to determine the optimum cut-off value for the division of low or high LINC01503 expression. Survival curves were generated using the Kaplan–Meier method and compared using the log-rank test. Univariate and multivariate Cox regression analyses were performed to test independent prognostic factors. A *p* value < 0.05 was considered statistically significant.

## Supplementary information

Supplemental Figure and Table Legends

Supplementary figure 1

Supplementary figure 2

Supplementary figure 3

Supplementary figure 4

Supplementary figure 5

Supplementary figure 6

Supplemental Table S1

Supplemental Table S2

Supplemental Table S3

Supplemental Table S4

Supplemental Table S5
