# Correction: AR-induced long non-coding RNA LINC01503 facilitates proliferation and metastasis via the SFPQ-FOSL1 axis in nasopharyngeal carcinoma

**DOI:** 10.1038/s41388-021-02050-7

**Published:** 2021-10-11

**Authors:** Shi-Wei He, Cheng Xu, Ying-Qing Li, Ying-Qin Li, Yin Zhao, Pan-Pan Zhang, Yuan Lei, Ye-Lin Liang, Jun-Yan Li, Qian Li, Yang Chen, Sheng-Yan Huang, Jun Ma, Na Liu

**Affiliations:** Sun Yat-sen University Cancer Center, State Key Laboratory of Oncology in South China, Collaborative Innovation Center of Cancer Medicine, Guangdong Key Laboratory of Nasopharyngeal Carcinoma Diagnosis and Therapy, No. 651 Dongfeng Road East, Guangzhou, 510060 Guangdong China

**Keywords:** Head and neck cancer, Cell migration, Prognostic markers

Correction to: *Oncogene* (2020) 39:5616–5632, 10.1038/s41388-020-01388-8, published online 13 July 2020

Following the publication of the above article, the authors noted two errors in Fig. 2 and Supplementary Fig. 2, in which the images of HK1 shCtrl group in Fig. 2g and 5-8F 1503 OE group at 0 h in Supplemental Fig. 2 were misused and have been replaced. The authors confirm that the mistakes do not affect the results and conclusions of the study and apologize for any inconvenience caused by this mistake. The corrected figures were provided below.

**Fig. 2:**
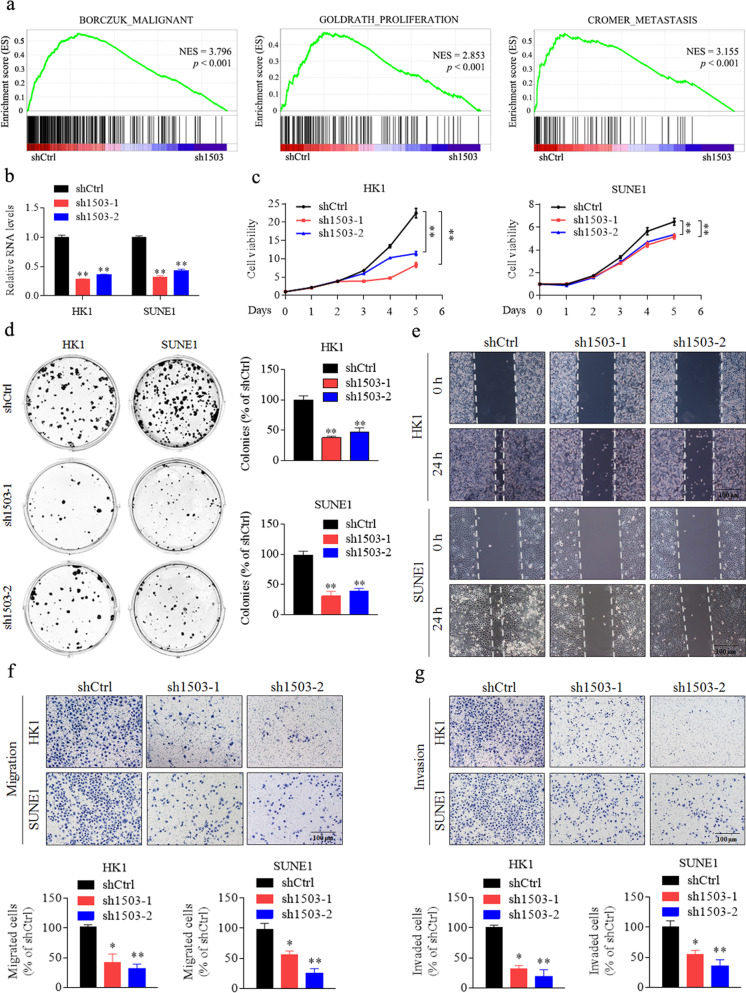
Silencing of LINC01503 inhibits NPC cell growth, migration, and invasion. **a** Malignant, proliferation-related, and metastasis-related biological functions were enriched by gene set enrichment analysis (GSEA) in HK1 cells transfected with LINC01503 shRNA (sh1503) or shCtrl. NES normalized enrichment score. FDR < 0.25, *p* < 0.001. **b** Relative expression of LINC01503 upon specific shRNA knockdown in HK1 and SUNE1 cells. **c** LINC01503 knockdown inhibited the cell growth of HK1 and SUNE1 cells as tested by CCK-8 assays. **d** LINC01503 knockdown decreased cellular survival effects as evaluated by colony formation assays. **e** LINC01503 knockdown inhibited the cellular movement ability of HK1 and SUNE1 cells as assessed by wound-healing assays. Scale bar, 100 μm. **f**, **g** LINC01503 knockdown inhibited the migration and invasion ability of HK1 and SUNE1 cells as determined by Transwell assays. Scale bar, 100 μm. Data are presented as the mean ± SD; *p* values were calculated with Student’s *t*-test; *p < 0.05, **p < 0.01.

Supplemental Fig. 2: Overexpression of LINC01503 promotes NPC cell growth, migration, and invasion in vitro. (a) Relative expression of LINC01503 in 5-8F and HONE1 cells transfected with LINC01503-expressing plasmid and empty vector. (b) LINC01503 overexpression promoted cell growth of 5-8F and HONE1 cells as shown by CCK-8 assays. (c) LINC01503 overexpression facilitated cellular survival effects as evaluated by colony formation assays. (d) LINC01503 overexpression accelerated the movement of 5-8F and HONE1 cells as assessed by wound healing assays. Scale bar, 100 μm. (e) LINC01503 overexpression promoted the migration and invasion ability of 5-8F and HONE1 cells as determined by transwell assays. Scale bar, 100 μm. * *p* < 0.05, ** *p* < 0.01.

The original article has been corrected.

## Supplementary information

Supplementary Fig. 2

