# Supplementary material for: AR-induced long non-coding RNA LINC01503 facilitates proliferation and metastasis via the SFPQ-FOSL1 axis in nasopharyngeal carcinoma
Source: Oncogene. 2020 Jul 13;39(34):5616–32. doi: 10.1038/s41388-020-01388-8 (PMC7441053; doi:10.1038/s41388-020-01388-8)
Supplement: Supplementary file 1 — Supplemental Figure and Table Legends [file 41388_2020_1388_MOESM1_ESM.docx]

**Supplemental Figure and Table Legends**

**Supplemental Fig. 1 GO and KEGG analysis of dysregulated genes in HK1 cells with LINC01503 knockdown.** (a-c) The top ten significantly enriched GO terms including Biological Process (a), Molecular Function (b) and Cellular Component (c) after LINC01503 knockdown; fold change >1.5; *p*-value <0.01. (d) The top ten significantly enriched KEGG pathway after LINC01503 knockdown. Fold change >1.5, *p*-value <0.01.

**Supplemental Fig. 2 Overexpression of LINC01503 promotes NPC cell growth, migration and invasion *in vitro*.** (a) Relative expression of LINC01503 in 5-8F and HONE1 cells transfected with LINC01503-expressing plasmid and empty vector. (b) LINC01503 overexpression promoted cell growth of 5-8F and HONE1 cells as shown by CCK-8 assays. (c) LINC01503 overexpression facilitated cellular survival effects as evaluated by colony formation assays. (d) LINC01503 overexpression accelerated the movement of 5-8F and HONE1 cells as assessed by wound healing assays. Scale bar, 100 μm. (e) LINC01503 overexpression promoted the migration and invasion ability of 5-8F and HONE1 cells as determined by transwell assays. Scale bar, 100μm. * *p*<0.05, ** *p* <0.01.

**Supplemental Fig. 3 Relative expression of LINC01503 and FOSL1 in the rescued experiments.** (a) The mRNA expression of LINC01503 and FOSL1 in HK1 and SUNE1 cells co-transfected with sh1503 or shCtrl plasmid in together with FOSL1-expressing plasmid or empty vector as measured by RT-qPCR assays. (b) The protein level of FOSL1 in the above treated cells was monitored by western blot.

**Supplemental Fig. 4 The expression of LINC01503 and FOSL1 in the formed tumor tissues from mice.** (a) LINC01503 expression was measured by *in situ* hybridization assay in the formed tumor tissues of mice. Scale bar, 100μm. FOSL1 expression was measured by IHC in the formed tumor tissues of mice. Scale bar, 100μm. (b) Ki67, SFPQ and FOSL1 expression was measured by IHC in the formed tumor tissues from mice. Scale bar, 100 μm.

**Supplemental Fig. S5 The expression of AR protein in knockdown or ectopic expression of AR in NPC cells.** (A) The protein expression of AR in HK1 and SUNE1 cells transfected with shAR or shCtrl plasmid as measured by Western blot. (B) The protein expression of AR in HK1 and SUNE1 cells transfected with AR-expressing plasmid or empty vector, as monitored by Western blot. (c-d) The expression of LINC01503 and its target FOSL1 were increased after DHT treatment, while decreased upon Enz treatment, as monitored by RT-qPCR and WB. (e) Enz-inhibited FOSL1 expression was reversed upon ectopic LINC01503 expression in HK1 and SUNE1 cells, as determined by RT-qPCR and WB.

**Supplemental Fig. 6 Full unedited Western blot gels for all figures.**

**Supplemental Table 1 Relationship between LINC01503 expression and clinical characteristics of NPC patients.**

**Supplemental Table 2 Cox regression analysis between LINC01503 expression and survival in NPC patients.**

**Supplemental Table 3 The top10 proteins found by mass spectrometry analysis in the LINC01503 sense and anti-sense group.**

**Supplemental Table 4 Transcription factors for the promoter of LINC01503.**

**Supplemental Table 5 Primers for RT-qPCR, vector construction, ChIP-qPCR and siRNAs.**
