# Supplementary figures and images for: AR-induced long non-coding RNA LINC01503 facilitates proliferation and metastasis via the SFPQ-FOSL1 axis in nasopharyngeal carcinoma
Source: Oncogene. 2020 Jul 13;39(34):5616–32. doi: 10.1038/s41388-020-01388-8 (PMC7441053; doi:10.1038/s41388-020-01388-8)

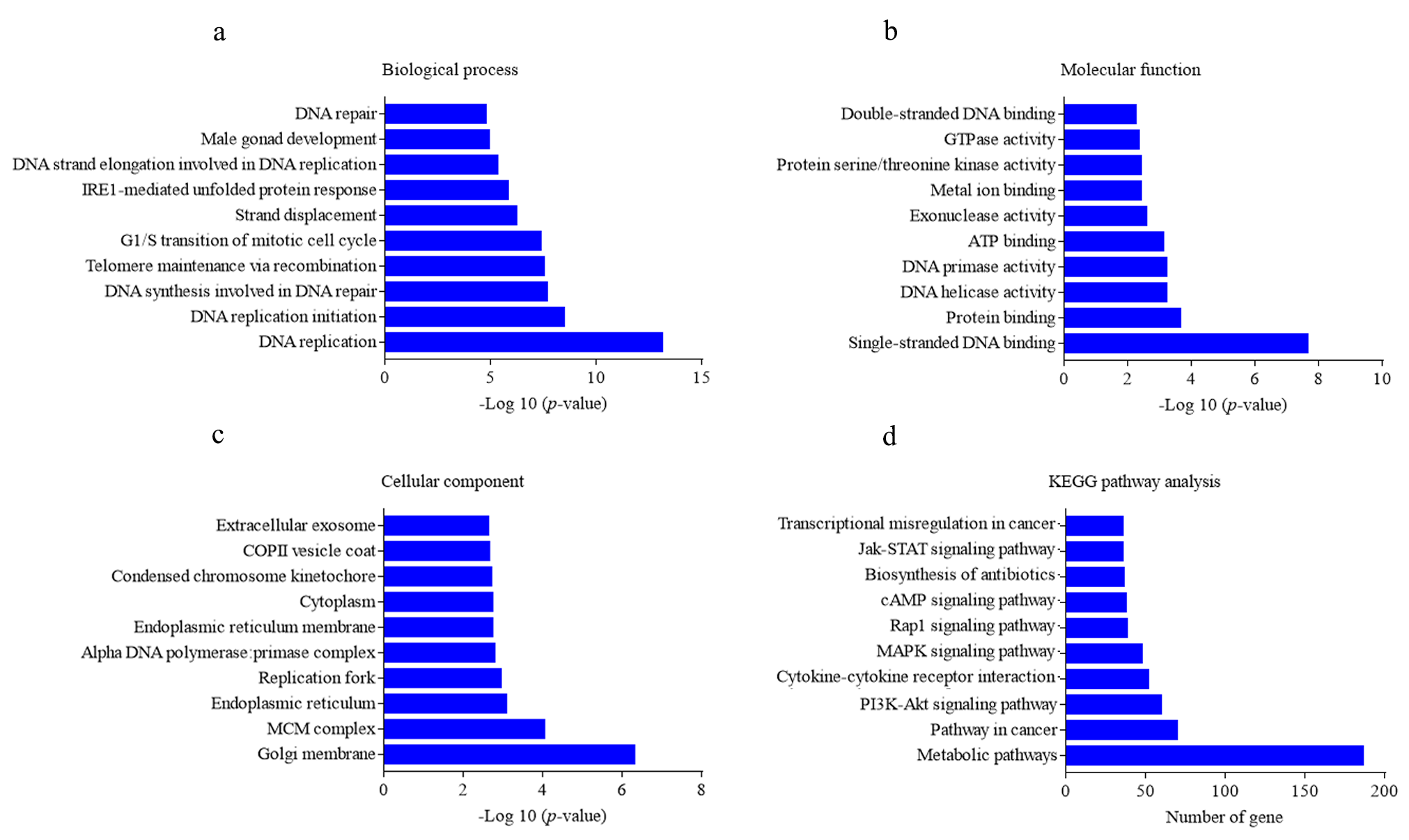

Supplement: Supplementary file 2 — Supplementary figure 1 [file 41388_2020_1388_MOESM2_ESM.tif]

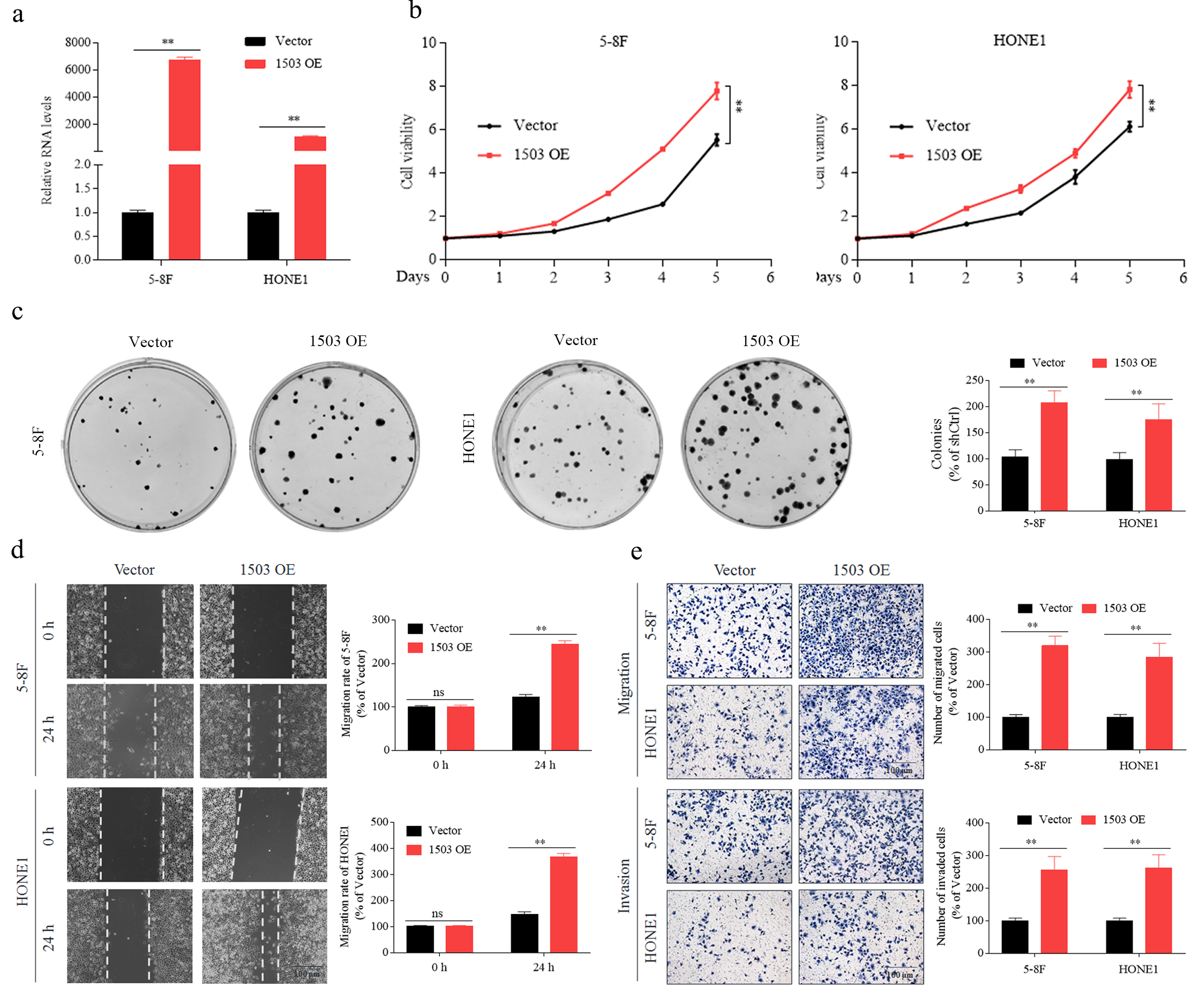

Supplement: Supplementary file 3 — Supplementary figure 2 [file 41388_2020_1388_MOESM3_ESM.tif]

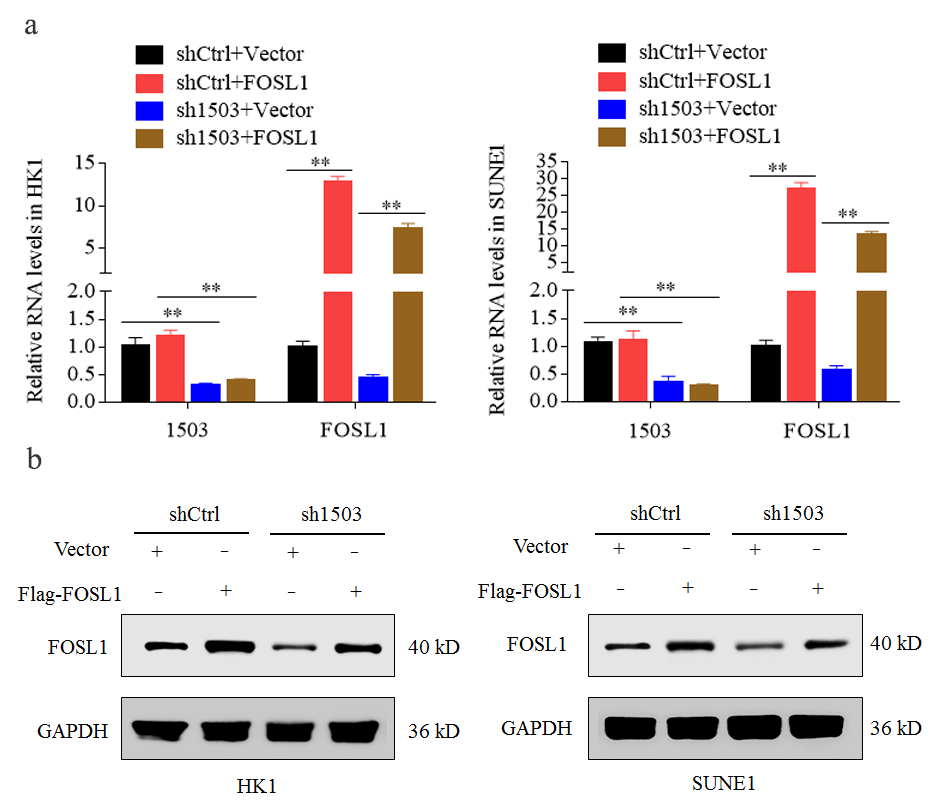

Supplement: Supplementary file 4 — Supplementary figure 3 [file 41388_2020_1388_MOESM4_ESM.tif]

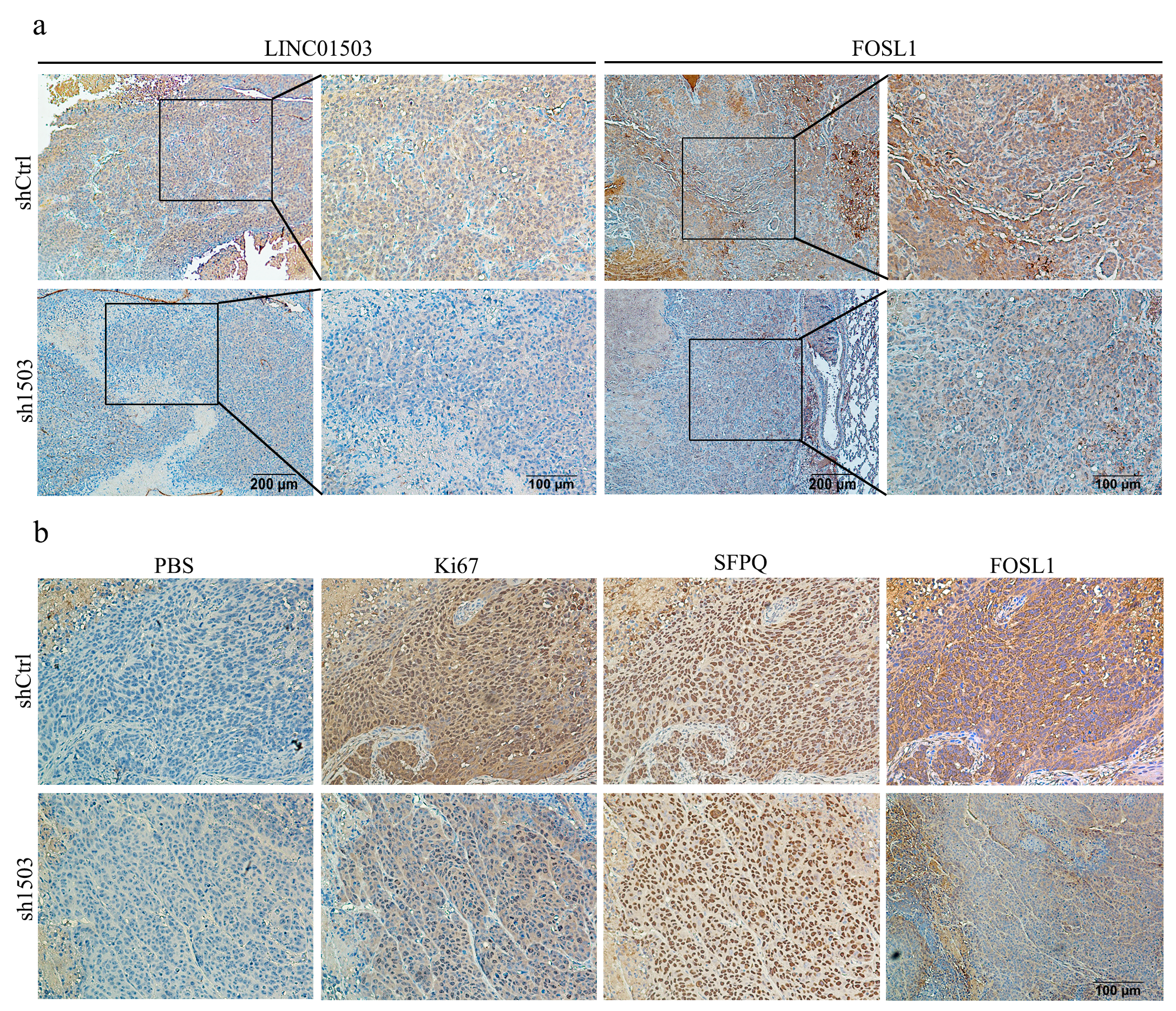

Supplement: Supplementary file 5 — Supplementary figure 4 [file 41388_2020_1388_MOESM5_ESM.tif]

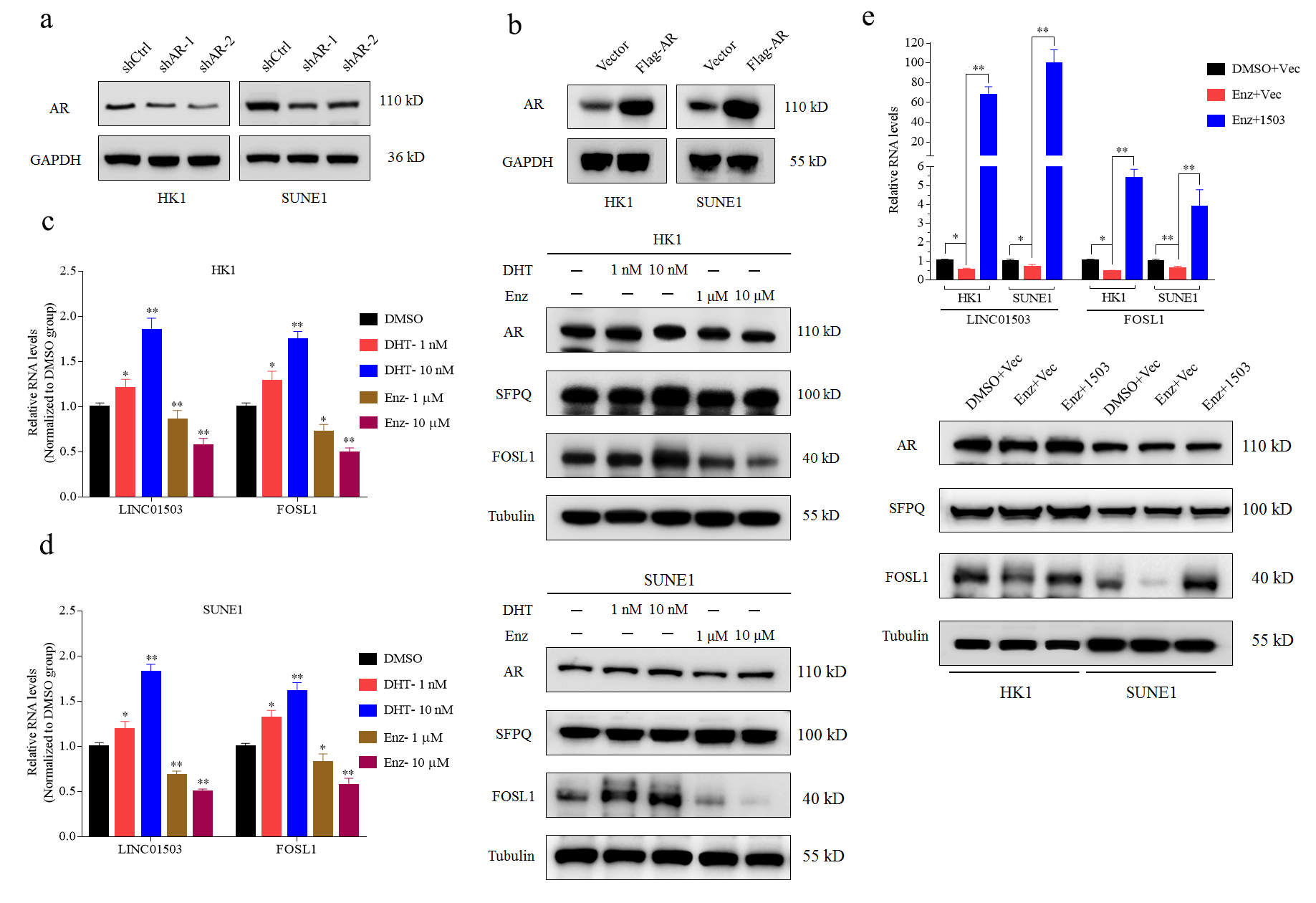

Supplement: Supplementary file 6 — Supplementary figure 5 [file 41388_2020_1388_MOESM6_ESM.tif]

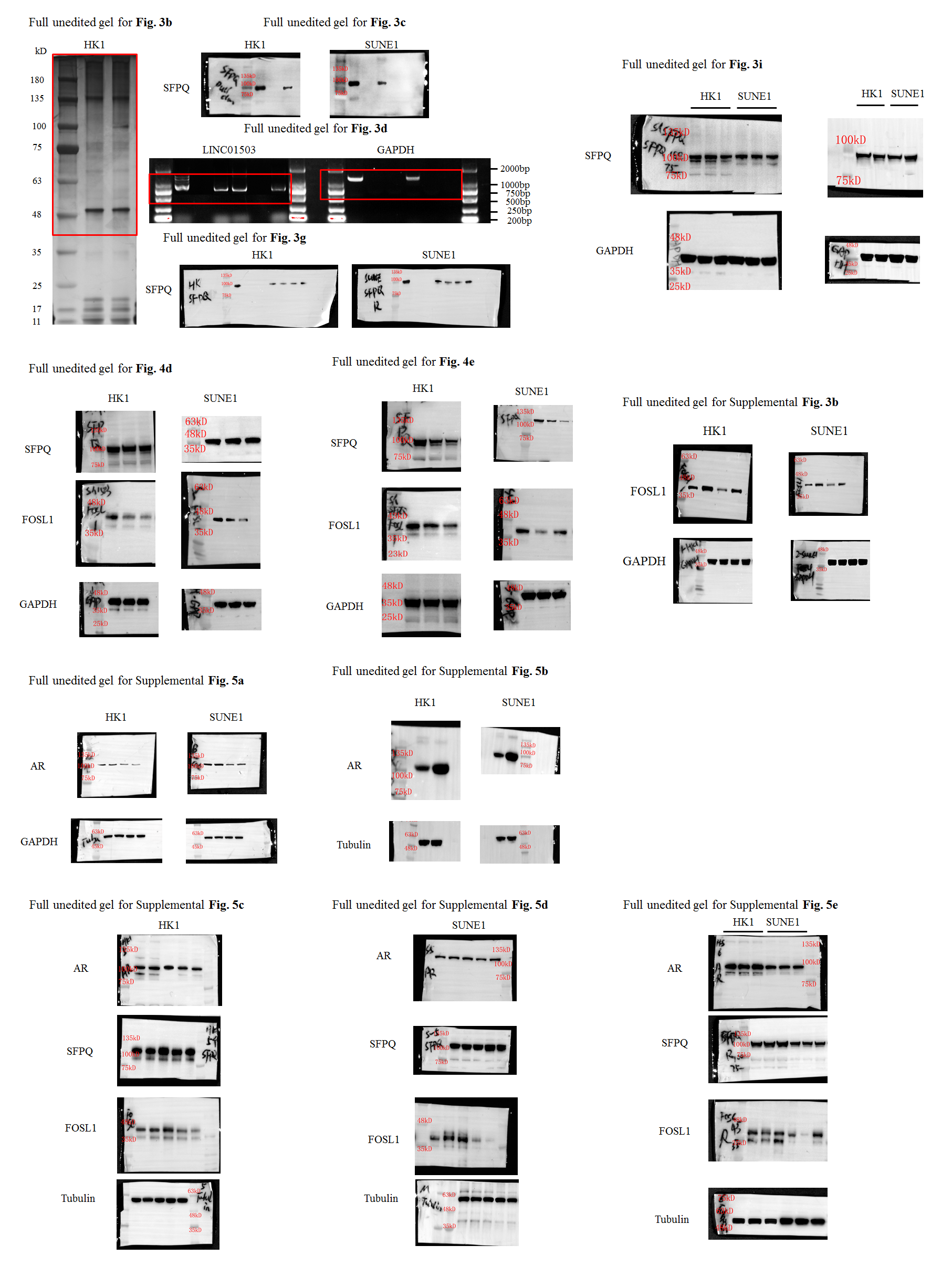

Supplement: Supplementary file 7 — Supplementary figure 6 [file 41388_2020_1388_MOESM7_ESM.tif]
