# Supplementary material for: AR-induced long non-coding RNA LINC01503 facilitates proliferation and metastasis via the SFPQ-FOSL1 axis in nasopharyngeal carcinoma
Source: Oncogene. 2020 Jul 13;39(34):5616–32. doi: 10.1038/s41388-020-01388-8 (PMC7441053; doi:10.1038/s41388-020-01388-8)
Supplement: Supplementary file 8 — Supplemental Table S1 [file 41388_2020_1388_MOESM8_ESM.docx]

**Supplemental Table S1 Relationship between LINC01503 expression and clinical characteristics of NPC patients.**

| **Variable** | **LINC01503** | | ***p*-value** |
| --- | --- | --- | --- |
|  | **Low level (n, %)**  **N=59** | **High level (n, %)**  **N=155** |  |
| **Age** |  |  |  |
| ≤ 45 years | 33 (55.9) | 76 (49.0) | 0.364 |
| >45 years | 26 (44.1) | 79 (51.0) |  |
| **Gender** |  |  |  |
| Female | 18 (30.5) | 29 (18.7) | 0.062 |
| Male | 41 (69.5) | 126 (81.3) |  |
| **VCA-IgA** |  |  |  |
| < 1:80 | 11 (18.6) | 27 (17.4) | 0.834 |
| ≥ 1:80 | 48 (81.4) | 128 (82.6) |  |
| **EA-IgA** |  |  |  |
| < 1:10 | 11 (18.6) | 42 (27.1) | 0.201 |
| ≥ 1:10 | 48 (81.4) | 113 (72.9) |  |
| **T Stage** |  |  |  |
| T1-T3 | 49 (83.1) | 124 (80.0) | 0.612 |
| T4 | 10 (16.9) | 31 (20.0) |  |
| **N Stage** |  |  |  |
| N0-N1 | 34 (57.6) | 76(49.0) | 0.261 |
| N2-N3 | 25 (42.4) | 79 (51.0) |  |
| **TNM Stage** |  |  |  |
| III | 42 (71.2) | 94 (60.6) | 0.181 |
| IV | 17 (29.8) | 61 (39.4) |  |

Abbreviations: VCA-IgA, viral capsid antigen immunoglobulin A; EA-IgA, early antigen immunoglobulin A; HR, hazard ratio.*p-*value was determined by χ^2^ or Fisher’s exact test.
