# Supplementary material for: AR-induced long non-coding RNA LINC01503 facilitates proliferation and metastasis via the SFPQ-FOSL1 axis in nasopharyngeal carcinoma
Source: Oncogene. 2020 Jul 13;39(34):5616–32. doi: 10.1038/s41388-020-01388-8 (PMC7441053; doi:10.1038/s41388-020-01388-8)
Supplement: Supplementary file 9 — Supplemental Table S2 [file 41388_2020_1388_MOESM9_ESM.docx]

**Supplemental Table S2 Cox regression analysis of LINC01503 expression and survival in NPC patients.**

| **Variables** | **Univariate analysis** | | |  | **Multivariable analysis** | | |
| --- | --- | --- | --- | --- | --- | --- | --- |
|  | **HR** | **95%CI** | ***p-value*** |  | **HR** | **95%CI** | ***p-value*** |
| **Overall survival** | | | | | | | |
| LINC01503level (high vs. low) | 0.34 | 0.17-0.68 | **0.002** |  | 0.34 | 0.17-0.68 | **0.003** |
| TNM Stage (IV vs. III) | 1.98 | 1.22-3.23 | **0.006** |  | 1.67 | 1.24-3.26 | **0.005** |
| Age (≥ 45 vs. <45 years) | 1.27 | 0.77-2.08 | 0.349 |  |  |  |  |
| Gender (Male vs. female) | 0.44 | 0.21-0.94 | 0.089 |  |  |  |  |
| VCA IgA (≥ 1:80 vs. < 1:80) | 1.00 | 0.99-1.01 | 0.545 |  |  |  |  |
| EA IgA (≥ 1:10 vs. < 1:10) | 0.99 | 0.99-1.01 | 0.804 |  |  |  |  |
| **Disease-free survival** | | | | | | | |
| LINC01503level (high vs. low) | 0.34 | 0.16-0.72 | **0.005** |  | 0.34 | 0.16-0.72 | **0.005** |
| TNM Stage (IV vs. III) | 1.74 | 1.05-2.87 | **0.030** |  | 1.69 | 1.03-2.77 | **0.038** |
| Age (≥ 45 vs. <45 years) | 1.29 | 0.78-2.15 | 0.323 |  |  |  |  |
| Gender (Male vs. female) | 0.42 | 0.19-0.92 | 0.058 |  |  |  |  |
| VCA IgA (≥ 1:80 vs. < 1:80) | 1.00 | 0.99-1.00 | 0.605 |  |  |  |  |
| EA IgA (≥ 1:10 vs. < 1:10) | 0.99 | 0.99-1.01 | 0.797 |  |  |  |  |
| **Distant metastasis-free survival** | | | | | | | |
| LINC01503level (high vs. low) | 0.40 | 0.18-0.91 | **0.028** |  | 0.40 | 0.18-0.89 | **0.025** |
| TNM Stage (IV vs. III) | 1.80 | 1.02-3.18 | **0.043** |  | 1.79 | 1.02-3.14 | **0.042** |
| Age (≥ 45 vs. <45 years) | 1.19 | 0.67-2.11 | 0.561 |  |  |  |  |
| Gender (Male vs. female) | 0.46 | 0.19-1.00 | 0.081 |  |  |  |  |
| VCA IgA (≥ 1:80 vs. < 1:80) | 1.00 | 0.99-1.01 | 0.709 |  |  |  |  |
| EA IgA (≥ 1:10 vs. < 1:10) | 0.99 | 0.99-1.01 | 0.538 |  |  |  |  |

VCA-IgA, viral capsid antigen immunoglobulin A; EA-IgA, early antigen immunoglobulin A; HR, hazard ratio. *p-*values werecalculated by univarate or multivariate Cox regression analysis.
