# Supplementary material for: AR-induced long non-coding RNA LINC01503 facilitates proliferation and metastasis via the SFPQ-FOSL1 axis in nasopharyngeal carcinoma
Source: Oncogene. 2020 Jul 13;39(34):5616–32. doi: 10.1038/s41388-020-01388-8 (PMC7441053; doi:10.1038/s41388-020-01388-8)
Supplement: Supplementary file 10 — Supplemental Table S3 [file 41388_2020_1388_MOESM10_ESM.docx]

**Supplemental Table S3 The top10 proteins found by mass spectrometry analysis in the LINC01503 sense and anti-sense group**

| Protein | Score | |
| --- | --- | --- |
| LINC01503sense group | | |
| Prelamin-A/C, LMNA | | 337 |
| Pyruvate carboxylase, PC | | 222 |
| **Splicing Factor Proline And Glutamine Rich, SFPQ** | | **113** |
| Non-POU domain-containing octamer-binding protein, NONO | | 111 |
| Y-box-binding protein 3, YBX3 | | 65 |
| ATP synthase subunit alpha, ATP5F1A | | 55 |
| Eukaryotic initiation factor, EIF4A1 | | 50 |
| Prohibitin-2, PHB2 | | 47 |
| High mobility group protein, HMGA1 | | 40 |
| Transcription elongation factor SPT5, SUPT5H | | 34 |
| LINC01503 anti-sense group | | |
| Methylcrotonoyl-CoA carboxylase beta chain, MCCC2 | | 349 |
| Putative elongation factor 1-alpha-like 3, EEF1A1P5 | | 308 |
| Xylosidexylosyltransferase 1, XXYLT1 | | 294 |
| Propionyl-CoA carboxylase alpha chain, PCCA | | 265 |
| Propionyl-CoA carboxylase beta chain, PCCB | | 202 |
| Serum albumin, ALB | | 146 |
| Protein Shroom3, SHROOM3 | | 129 |
| Pyruvate kinase, PKM | | 114 |
| Lipoamideacyltransferase component of branched-chain alpha-keto  acid dehydrogenase complex, DBT | | 89 |
| ADP/ATP translocase 2, SLC25A5 | | 73 |
