# Supplementary material for: AR-induced long non-coding RNA LINC01503 facilitates proliferation and metastasis via the SFPQ-FOSL1 axis in nasopharyngeal carcinoma
Source: Oncogene. 2020 Jul 13;39(34):5616–32. doi: 10.1038/s41388-020-01388-8 (PMC7441053; doi:10.1038/s41388-020-01388-8)
Supplement: Supplementary file 11 — Supplemental Table S4 [file 41388_2020_1388_MOESM11_ESM.docx]

**Supplemental Table S4 Transcription factors for the promoter of LINC01503**

| Matrix ID | Name | Score | Relative score | Start | End | Predicted sequence |
| --- | --- | --- | --- | --- | --- | --- |
| MA0007.2 | AR | 9.09422 | 0.86467 | 1234 | 1248 | tagaacaatgtgtag |
| MA0007.2 | AR | 7.72889 | 0.846303 | 1452 | 1466 | aagaacacaggctct |
| MA0634.1 | ALX3 | 7.41299 | 0.868539 | 1967 | 1976 | ccccattaaa |
| MA0634.1 | ALX3 | 6.44243 | 0.842787 | 1212 | 1221 | accaataaca |
| MA0634.1 | ALX3 | 6.33051 | 0.839817 | 116 | 125 | tgccattaac |
| MA0634.1 | ALX3 | 6.30677 | 0.839188 | 420 | 429 | aaaaattagc |
| MA0007.2 | AR | 6.29964 | 0.827077 | 1394 | 1408 | aagatcacacagctc |
| MA0634.1 | ALX3 | 6.09648 | 0.833608 | 1254 | 1263 | ctcaataaat |
| MA0634.1 | ALX3 | 5.63153 | 0.821271 | 420 | 429 | gctaattttt |
| MA0634.1 | ALX3 | 4.87578 | 0.801219 | 1903 | 1912 | acatattaaa |
