# Supplementary material for: AR-induced long non-coding RNA LINC01503 facilitates proliferation and metastasis via the SFPQ-FOSL1 axis in nasopharyngeal carcinoma
Source: Oncogene. 2020 Jul 13;39(34):5616–32. doi: 10.1038/s41388-020-01388-8 (PMC7441053; doi:10.1038/s41388-020-01388-8)
Supplement: Supplementary file 12 — Supplemental Table S5 [file 41388_2020_1388_MOESM12_ESM.docx]

**Supplemental Table S5 Primers for RT-qPCR, vector construction, ChIP-qPCR and siRNAs**

| **Name** | **Sequences (5'-3')** |
| --- | --- |
| **Primers for RT-qPCR from freshly-frozen tissues and cells** | |
| LINC01503-F | CTTTCCCTGAGGACCATCTG |
| LINC01503-R | CAAAATCCGGTCTTTCTGGA |
| SFPQ-F | TCAGGCAAATCTTTTGCGCC |
| SFPQ-R | CGTTCCTCCTCTTGCCTCAA |
| FOSL1-F | CATGAGTGGCAGTCAGGAGC |
| FOSL1-R | CCGGGCTGATCTGTTCACAAG |
| ALX3-F | AGAGACGGGCCCTCTAACTT |
| ALX3-R | TGGAACCAGACCTGTACCCG |
| AR-F | TTCCCTTCAGCGGCTCTTTT |
| AR-R | GACATGCGTTTGGAGACTGC |
| GAPDH-F | AACGGATTTGGTCGTATTGG |
| GAPDH-R | TTGATTTTGGAGGGATCTCG |
| **Primers for RT-qPCR from FFPE tissues** | |
| LINC01503- F | ACTGTGCACTCTTCGTGTTCA |
| LINC01503-R | CCTTAAACACCCATCCGGGG |
| GAPDH-F | GCTAGGGACGGCCTGAAG |
| GAPDH-R | GCCCAATACGACCAAATCCGT |
| **shRNA sequences** | |
| sh1503-1 | GCTCGGAATACCCACCTTTCT |
| sh1503-2 | GCCTCTGACAAGTGTGTACCT |
| shSFPQ-1 | GGAAGAAGCCTTTAGCCAATT |
| shSFPQ-2 | GGAAGATGCCTATCATGAACA |
| shFOSL1-1 | GGATGGTACAGCCTCATTTCC |
| shFOSL1-2 | GGAGACTGACAAACTGGAAGA |
| shALX3-1 | GCACTGGTGTCTGACACTAAG |
| shALX3-2 | GCAGAAATTCCACCAGAAATC |
| shAR-1 | GCTACTCTTCAGCATTATTCC |
| shAR-2 | GCACCTTATGTCCTCCCTTCA |
| **Primers for plasmid constructs** | |
| 1503-F | CGCGGATCCGGCTGCACAGTCACACGC |
| 1503-R | CCGCTCGAGTGCTGAAAGAAACTCATTGC |
| 1503 M1 R | CGCGGATCCACACGCGGCGCCCCATTA |
| 1503 M1 F | CCGCTCGAGCCTGACACGTAGGTACACAC |
| 1503 M2 F | CGCGGATCCGGGCTCGGAATACCCACCT |
| 1503 M2 R | CCGCTCGAGTCTGGAGAGGAGACCTAGGG |
| 1503 M3 F | CGCGGATCCCCCATCCTTCCTGCAGGCCTT |
| 1503 M3 R | CCGCTCGAGTCATTGCATCGTGTTTTTAA |
| 1503 M4 F | CGCGGATCCCCCATCCTTCCTGCAGGCCTT |
| 1503 M4 R | CCGCTCGAGTCTGGAGAGGAGACCTAGGG |
| SFPQ-F | CGCACGCGTATGTCTCGGGATCGGTTCCGGAGTCGTGGC |
| SFPQ-R | CCGCTCGAGAAATCGGGGTTTTTTGTTTGGGCCTTCGTACTCT |
| FOSL1-F | CGCGGATCCATGTTCCGAGACTTCGGGGAACC |
| FOSL1-R | CCGCTCGAGCAAAGCGAGGAGGGTTGGAGAGC |
| ALX3-F | CGCGGATCCATGGACCCCGAGCACTGCGC |
| ALX3-R | CCGCTCGAGCGTGGTCCAGTTCAGAAGGC |
| AR-F | CGCGGATCCATGATACTCTGGCTTCACAGTT |
| AR-R | CCGCTCGAGCTGGGTGTGGAAATAGATGGGC |
| FOSL1 WT-F | CGGACGCGTGCACGGGTGTTCTGTCCACGGAT |
| FOSL1 WT-R | CCGCTCGAGGCTGGGCTCTCCTGCTTCTCCAT |
| FOSL1 Mut-F | CGGACGCGTGCATTACCTTATCGCAAACA |
| FOSL1 Mut-R | CCGCTCGAGTCGAGGCCTGGGCTAACCTC |
| 1503 WT-F | CGGACGCGTGACAGGCTGTGGGCAGCCCTCA |
| 1503 WT-R | CCGCTCGAGGGTCCTTCCCGGCCATGGAGG |
| 1503 Mut1-F | CGGACGCGTACCACTTCCCACTGCCCACTG |
| 1503 Mut1-R | CCGCTCGAGGCTCTTGTTGCCCACGCTGGA |
| 1503 Mut-2-F | CGGACGCGTAAATGAATGAAGGCCAAACTT |
| 1503 Mut-2-R | CCGCTCGAGGATTCTCCTGCCTCAGCCTCC |
| **Primers for LINC01503 sense and antisense** | |
| 1503 sense-F | CGCGGATCCGGCTGCACAGTCACACGC |
| 1503 sense-R | CCGCTCGAGTGCTGAAAGAAACTCATTGC |
| 1503 anti-sense-F | CGCGGATCCTGCTGAAAGAAACTCATTGC |
| 1503 anti-sense-R | CCGCTCGAGGGCTGCACAGTCACACGC |
| **Primers for RIP-PCR** | |
| 1503-RIP F | CTTTCCCTGAGGACCATCTG |
| 1503-RIP R | CTTTCCCTGAGGACCATCTG |
| GAPDH-RIP F | tcttgactcaccctgccct |
| GAPDH-RIP R | acaaaggcactcctggaaac |
| **Primers for ChIP-PCR** | |
| FOSL1-F | CACTCTCCTCTCCTTTCTCT |
| FOSL1-R | AAGTGAGATCACAGATGTGA |
| 1503-1-F | AGAACACAGCTAGCAGTTCC |
| 1503-1-R | GGCTCATTGCCTGGCATGTA |
| 1503-2-F | AAGTGCTTTACAAGGCTGAC |
| 1503-2-R | AGCCAGGTGCGAAGAAGGAT |
| **Probe sequences for LINC01503** | |
| 1503 probe 1 | GAGTAGTTCTTTAATCTGCTTATCTGGTGATTCCATCACA |
| 1503 probe 2 | GGCTTTCTAGCCTGATGTGAGTTGTGTTTACACACATATA |
| 1503 probe 3 | GCAATCTGTTTAATGTGCCAGTTGTCTCTGTGAGTCCTC |
