# Supplementary figures and images for: Correction: AR-induced long non-coding RNA LINC01503 facilitates proliferation and metastasis via the SFPQ-FOSL1 axis in nasopharyngeal carcinoma
Source: Oncogene. 2021 Oct 11;40(49):6703–4. doi: 10.1038/s41388-021-02050-7 (PMC8660634; doi:10.1038/s41388-021-02050-7)

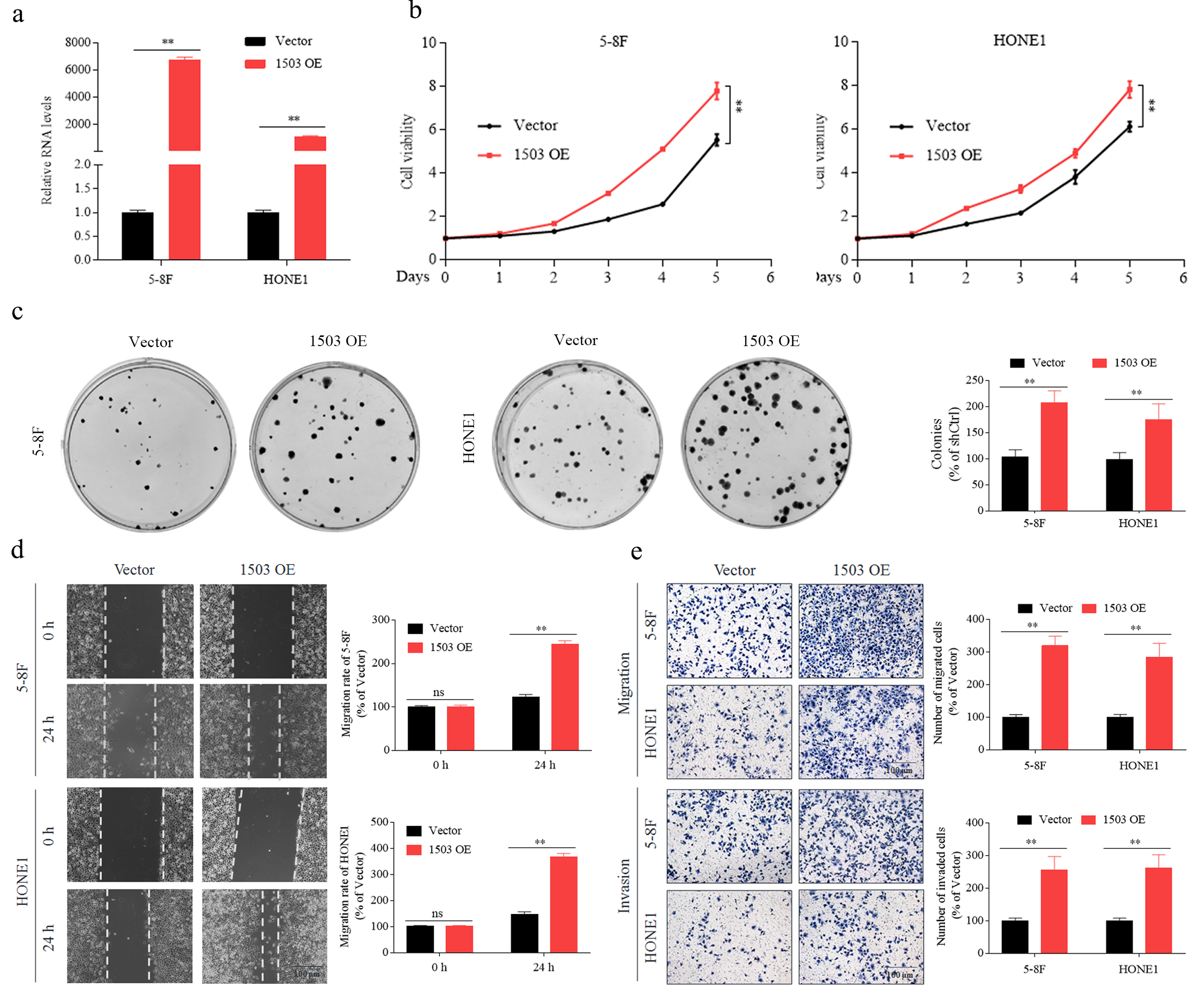

Supplement: Supplementary file 1 — Supplementary Fig. 2 [file 41388_2021_2050_MOESM1_ESM.tif]
